# Multimodal feature fusion model for breast mass malignant risk stratification

**DOI:** 10.3389/fonc.2026.1782135

**Published:** 2026-06-03

**Authors:** Shengxin Pei, Xiumei Tang, Hongxia Su, Jingyan Liu, Zihan Lan, Siyu Wang, Yulan Peng

**Affiliations:** 1 Department of Ultrasound, West China Hospital/West China School of Medicine, Sichuan University, Chengdu, China; 2 Department of Ultrasound Medicine, The First Hospital of Northwest University, Xi’an, Shaanxi, China; 3 Institute of Hospital Management, West China Hospital of Sichuan University, Chengdu, China; 4 State Key Laboratory of Respiratory Health and Multimorbidity, Chengdu, Sichuan, China; 5 West China School of Nursing, Sichuan University, Chengdu, China; 6 Department of Outpatient, West China Hospital of Sichuan University, Chengdu, China

**Keywords:** artificial intelligence, breast cancer, machine learning, multimodal data, risk stratification

## Abstract

**Introduction:**

This study aims to develop and validate machine learning models that integrate multimodal features (BI-RADS terminology, ultrasound imaging, and radiomics) to improve breast mass malignancy risk stratification and compare their diagnostic performance across different BI-RADS categories.

**Methods:**

This retrospective cohort study analyzed data from 2, 685 patients with 3, 703 ultrasound images collected from July 2019 to March 2024 at a single medical center. Patients included women with complete ultrasound images and clear pathological diagnoses. The dataset comprised 2, 069 benign cases (2, 762 images) and 616 malignant cases (941 images), randomly divided into training (n=2, 979 images) and validation (n=724 images) sets. Primary outcomes were diagnostic accuracy and area under the receiver operating characteristic curve (AUC) for distinguishing malignant from benign breast masses. Three machine learning models (Logistic Regression, Support Vector Machine, and Random Forest) were trained using BI-RADS terminology features, ultrasound quantitative features, radiomics features, and combined multimodal features. Performance was evaluated both overall and within specific BI-RADS subcategories (2, 3, 4a, 4b, 4c, and 5).

**Results:**

Among 2, 685 patients, the Random Forest model using combined multimodal features achieved the highest overall performance with an AUC of 0.850 (95% CI 0.810- 0.875). For single-modality approaches, Logistic Regression performed best with BI-RADS terminology features, with an AUC of 0.820 (95% CI, 0.775-0.856), and radiomics features, with an AUC of 0.740 (95% CI, 0.706-0.780); while Random Forest was optimal for ultrasound imaging features, with an AUC of 0.800 (95% CI, 0.768-0.839). Subgroup analysis revealed excellent performance for BI-RADS categories 2 (AUC, 1.000-1.000) and 3 (AUC, 0.947-0.957), acceptable performance for 5 (AUC, 0.813-0.870) and 4a (AUC, 0.800-0.867), but poor performance for categories 4b (AUC, 0.649-0.709), 4c (AUC, 0.551-0.623).

**Discussion:**

This study demonstrates that machine learning models integrating multimodal ultrasound features can effectively stratify breast mass malignancy risk, with the Random Forest model using combined features showing superior performance. The approach shows particular strength in BI-RADS categories 2, 3, 5 and 4a, suggesting potential clinical utility for reducing unnecessary biopsies and improving diagnostic confidence. However, performance limitations in higher-risk categories (4b, 4c) indicate need for further model refinement and multicenter validation before clinical implementation.

## 1 Introduction

Breast cancer (BC) is the most frequently diagnosed cancer in women and ranks second (after lung cancer) among causes of cancer-related death (1). It was reported that Chinese BC patients had the highest rates of morbidity and mortality worldwide (34.38% and 8.38%, respectively) (2). It is estimated that new cases of BC will surge at a rate of 3.2 million per year globally by 2050, with a tendency for patients to be much younger (3). Conventionally, the diagnosis of BC could be done by biopsy, breast magnetic resonance imaging (MRI), diagnostic mammogram, or breast ultrasound (BUS) (4). BUS is one of the typical medical imaging modalities with the advantages of less use of contrast agents, no energy rays, and suitability for all ages, and it has been applied in BC screening, diagnosing, and follow-up for over 60 years due to its convenient and non-invasiveness (5). Via assessing the morphology, orientation, internal structure, and margins of lesions from multiple high-resolution planes, BUS can be used to precisely diagnose predominantly fatty breasts and dense glandular structures (6, 7). Historically, the diagnosis of BC requires visual inspection of histopathology slides by experienced pathologists. And its limitations restrict large-scale application in clinical practice. (i) the diagnosis of BUS is highly operator-dependent, which may have a high inter-observer variation; (ii) the low definition/resolution rate makes BUS not suitable for small mass and atypical tissue; (iii) interpreting images is a tedious and time-consuming task; and (iv) numerous data increase the risk of oversight errors when in analysis (3, 8, 9). These disadvantages resulted in the low average sensitivity and specificity in BC clinical diagnosis (up to 70%) and called for more efficient and accurate methods to improve the situation.

In the rapidly evolving context of computer technologies and image processing software, advances in artificial intelligence (AI) technologies have demonstrated remarkable potential in oncology image-recognition tasks assisting physicians in accurate diagnosis (10). Machine learning can be used in pattern recognition to train prediction models and infer generalizations (11). Currently, classic approach, including logistic regression (LR), Support Vector Machine (SVM), Artificial Neural Networks (ANN), Decision Tree (DT), Random Forest (RF), Naive Bayes (NB), and K-Nearest Neighbor (KNN) are widely used in BUS image process (12).

Generally, sonographic features for determining BC diagnosis were mainly through Breast Imaging Reporting and Data System (BI-RADS) categories based on the radiologist’s interpretation (13). Microcosmic features of images, such as texture features, cannot be identified by visual interpretation (14). Features extracted from BUS images mainly involved characteristics of images, such as tumor size, shape, texture, and characteristics of radiomics, such as texture features, obtained by calculating the statistical correlation between the signal intensities of adjacent voxels. The applications of radiomics are an emerging field of research aiming to extract meaningful features from clinical images, especially in BC diagnosis, category classification, prognosis prediction, molecular characteristics identification, and treatment assessment (15, 16). Whereas the majority of radiomics studies were retrospective design, had a relatively small sample size, and often reported questionable or uncertain repeatability assessments.

Our primary goal was to develop and validate three traditional machine learning models including logistic regression (LR), support vector machine (SVM), and random forest (RF) using BI-RADS category terminology features, ultrasound image features, radiomics features, and their combinations via whole dataset and subgroup dataset (by BI-RADS category). Our secondary goal was to compare the accuracy and area under the receiver operating characteristic (ROC) curves (AUC) of these models to identify the optimal model in whole dataset. Furthermore, the optimal model will be tested within each subgroup dataset (by BI-RADS category), and we will assess the model’s accuracy and AUC in classifying subcategories of the BI-RADS classification.

## 2 Materials and methods

### 2.1 Instrument details

All breast ultrasound examinations were performed at the Department of Ultrasound, West China Hospital, using high-resolution ultrasound scanners equipped with linear array transducers(frequency range 5–14 MHz). Standard breast ultrasound protocol was followed according to American College of Radiology guidelines, including systematic evaluation in both transverse and longitudinal planes. Images were acquired in B-mode with standardized settings: imaging depth 3–5 cm adjusted based on lesion location, focal zone positioned at the lesion level, and time-gain compensation optimized for uniform tissue visualization. All images were stored in DICOM format with spatial resolution ≥300 DPI. Examinations were performed by certified sonographers with ≥5 years of experience in breast imaging, and all images were reviewed and interpreted by board-certified radiologists with ≥3 years of subspecialty training in breast imaging.

### 2.2 Study design, ethics, and setting

**Figure 1:**
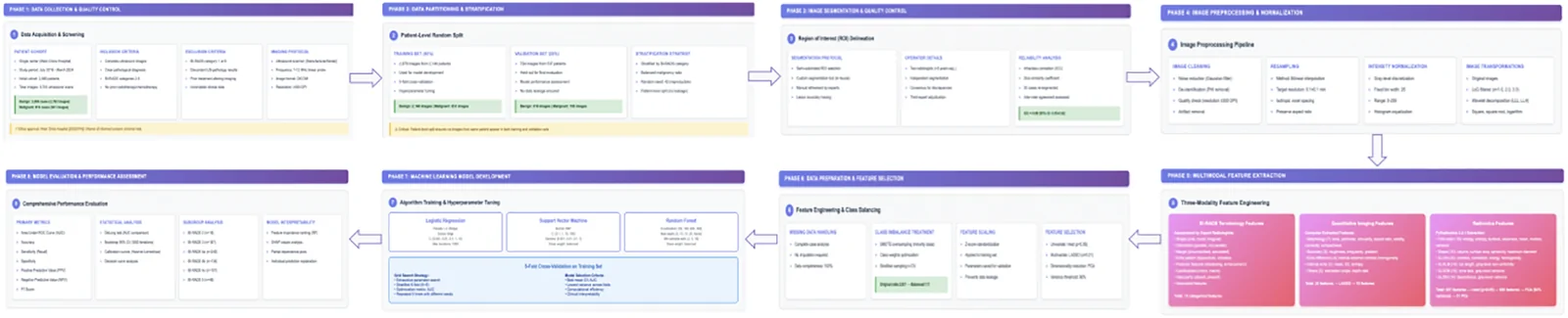
Technical pipeline for multimodal machine learning model development. This flowchart illustrates the complete technical pipeline from data acquisition to model evaluation. The study included 2, 685 patients with 3, 703 ultrasound images, divided into training (80%, n=2, 979) and validation (20%, n=724) sets using patient-level random split to prevent data leakage. Three feature modalities were extracted: (i) BI-RADS terminology features assessed by expert radiologists (n=15 features), (ii) quantitative imaging features computed from ultrasound images (n=20 to 15 features after LASSO selection), and (3) radiomics features extracted using PyRadiomics (n=837 to 600 to 31 principal components after t-test and PCA). Image preprocessing included noise reduction, resampling (0.1×0.1 mm), and intensity discretization (bin width=25). Class imbalance was addressed using SMOTE oversampling. Three machine learning algorithms (Logistic Regression, Support Vector Machine, Random Forest) were trained using 5-fold cross-validation with grid search for hyperparameter tuning. The optimal Random Forest model using combined multimodal features achieved an AUC of 0.850 (95% CI 0.810-0.875) on the validation set, outperforming single-modality approaches. Performance metrics included accuracy, sensitivity, specificity, PPV, NPV, and F1 score, with statistical comparisons conducted using DeLong test. Model interpretability was assessed using feature importance analysis and SHAP values. BI-RADS, Breast Imaging Reporting and Data System; LASSO, Least Absolute Shrinkage and Selection Operator; PCA, Principal Component Analysis; SMOTE, Synthetic Minority Over-sampling Technique; GLCM, Gray-Level Co-occurrence Matrix; GLRLM, Gray-Level Run Length Matrix; GLSZM, Gray-Level Size Zone Matrix; GLDM, Gray-Level Dependence Matrix; ROC, Receiver Operating Characteristic; AUC, Area Under the Curve; PPV, Positive Predictive Value; NPV, Negative Predictive Value; SHAP, SHapley Additive exPlanations.

This retrospective cohort study was approved by the Ethics Committee of West China Hospital [2022 (974)] with a waiver of informed consent because it was determined to be of minimal risk. The data was collected from July 2019 to March 2024. Cases were excluded if patients had received any prior radiotherapy or chemotherapy that could alter imaging characteristics, if there was discordance between ultrasonic and pathological results, or if lesions were classified as BI-RADS category 1 (normal) or category 6 (known malignancy). Only cases with clear pathological patterns from surgery specimens were included for model derivation and validation. This study included a unique cohort of 2, 685 patients with no overlap with previously published datasets. Each patient was included only once during the study period (July 2019 to March 2024), and no patient received multiple pathological diagnoses for the same lesion. For patients with multiple ultrasound images of the same lesion or bilateral breast masses, all images from the same patient were assigned exclusively to either the training set or the validation set to prevent data leakage. The inclusion and exclusion process can be seen in **Figure 1**.

### 2.3 Reporting guideline

We followed the Transparent Reporting of a Multivariable Prediction Model for Individual Prognosis or Diagnosis plus Artificial Intelligence (TRIPOD+AI) reporting guidelines (17) and adhered to the CheckList for EvaluAtion of Radiomics research (CLEAR) endorsed by the European Society of Radiology and European Society of Medical Imaging Informatics (18). A completed CLEAR checklist is provided in **Supplementary File**.

### 2.4 Outcome and predictors

The primary outcome of interest was breast mass malignancy status, defined as a binary outcome of malignant versus benign classification based on histopathological examination following biopsy or surgical resection. Pathological diagnosis served as the gold standard reference for all cases.

Model variables included BI-RADS standard terminology features assessed by expert radiologists with >3 years of experience, quantitative ultrasound imaging features, and radiomics features extracted from ultrasound images. BI-RADS terminology features encompassed morphological characteristics (shape, orientation, margins), internal echo patterns, calcification patterns, posterior acoustic features, vascularity, and associated features according to American College of Radiology standards. Quantitative imaging features included 20 computer-extracted characteristics describing tumor morphology (7 features), boundary characteristics (3 features), echo differences between internal and external regions (4 features), internal echo properties (3 features), and other quantitative measures (3 features), with 15 features retained after LASSO regression feature selection. Radiomics features comprised 837 high-throughput quantitative features extracted using PyRadiomics, including first-order statistical features (energy, kurtosis, entropy), morphological features (area, maximum diameter), texture features from gray-level co-occurrence matrix (GLCM), gray-level size zone matrix (GLSZM), gray-level dependence matrix (GLDM), and gray-level run length matrix (GLRLM), as well as features derived from filtered image transformations. Following T-test screening (*p* < 0.05), 600 statistically significant features were retained, which were further reduced to 31 principal components using principal component analysis to preserve 90% of the original data variance. The calculation formula and description of quantitative features for BUS images **Supplementary Table 1**. These measures were chosen for their clinical relevance, routine availability in breast ultrasound examinations, and prior evidence supporting their diagnostic utility. BI-RADS classifications were performed independently by two expert radiologists, with discrepancies resolved by a third senior radiologist to ensure consistency.

### 2.5 Sample size calculation

This retrospective study utilized all available cases meeting eligibility criteria during the study period (July 2019 to March 2024), yielding a convenience sample of 2, 685 patients with 3, 703 ultrasound images. The dataset was partitioned at the patient level into a training set (80%; 2, 148 patients with 2, 979 images, including 2, 146 benign and 833 malignant) and a held-out validation set (20%; 537 patients with 724 images, including 616 benign and 108 malignant). *Post-hoc* power analysis was conducted to verify the adequacy of the sample size for the primary outcome (malignant vs. benign classification). (i) For the validation set (537 patients contributing 724 images; 616 benign and 108 malignant), assuming an expected AUC of 0.85 (based on pilot data and prior literature) and a null hypothesis AUC of 0.50 (no discriminative ability), the achieved statistical power exceeded 99% (α = 0.05, two-sided) using the method of Hanley and McNeil (1982). The sample size also provided adequate power (>90%) to detect a clinically meaningful AUC difference of ≥0.05 between competing models using the DeLong test. (ii) For radiomics feature selection, the events-per-variable (EPV) ratio was calculated based on the number of malignant cases in the training set (n = 833), as recommended for predictive modeling studies. Prior to dimensionality reduction, 600 statistically significant radiomics features were retained after t-test screening, yielding an EPV of 1.4:1 (833/600), which was insufficient for direct model fitting. This justified the subsequent application of principal component analysis (PCA), which reduced the feature space to 31 principal components explaining 90% of the cumulative variance. After PCA reduction, the EPV ratio was 26.9:1 (833/31), well above the recommended minimum threshold of 10:1, ensuring adequate model stability and reducing overfitting risk. When considering the combined multimodal feature set (15 BI-RADS terminology features + 15 imaging features after LASSO selection + 31 radiomics principal components = 61 total input variables), the EPV ratio was 13.7:1 (833/61), still exceeding the 10:1 criterion. (iii) For subgroup analyses within BI-RADS categories, sample sizes ranged from 90 images (BI-RADS 2; 74 training, 16 validation) to 1, 217 images (BI-RADS 4a; 977 training, 240 validation). We acknowledge that smaller subgroups, particularly BI-RADS 2 (n = 90) and BI-RADS 5 (n = 239), may have limited statistical power for definitive conclusions regarding subgroup-specific model performance, and these results should be interpreted with caution. This limitation is further addressed in the Discussion section. Detailed patient-level data partition strategy can be seen in **Supplementary Table 2**.

### 2.6 Model development

For BI-RADS features, two expert radiologists (with > 3 years of work experience) participated in ultrasonic image evaluation and BI-RADS classification according to the standards of the American College of Radiology. If there was any controversy, the third radiologist solved the discrepancy and made the final decision.

For ultrasound image features, we used the following steps: (i) Image Cleaning and De-identification. Medical image was cleaned, and the noise was removed through image preprocessing operations. We eliminated images with low resolution, blurred, undisplay, or incomplete. Personal information is desensitized. (ii) Selection of the Region of Interest (ROI). Lesion segmentation was performed using a semi-automated approach combining manual boundary tracing with computer-assisted edge refinement. The workflow included manual delineation of the lesion on the image showing maximum diameter; automated boundary optimization using gradient-based edge detection; manual verification and adjustment. The ROI included the entire lesion with a 2-pixel margin, excluding normal surrounding tissue. The segmentation tool was developed in-house using Python 3.8 and OpenCV. (iii) Dataset Division. The dataset is randomly split into training and testing sets at a ratio of approximately 3:1. A 5-fold cross-validation method is used to optimize model performance. The process will be repeated 5 times, ensuring that each subset is used as validation data once. And the optimal algorithm for each feature set was determined by the highest mean cross-validation AUC. (iv) Feature Extraction. Features including dynamic, textural, and morphological were extracted via system developed by our team. A total of 20 features were described, including 7 morphological features, 3 boundary features, 4 echo differences, 3 internal echo features, and 3 other features. (v) Feature Selection. The Least Absolute Shrinkage and Selection Operator (LASSO) regression was applied to identify variables and corresponding regression coefficients that minimize prediction error. All segmentations were performed by two board-certified radiologists (8 years and 5 years of breast imaging experience, respectively), blinded to clinical and pathological information. Inter-observer reliability was assessed on 50 randomly selected cases, yielding excellent agreement (ICC = 0.89, 95% CI: 0.85-0.92 for first-order features; ICC = 0.86, 95% CI: 0.82-0.90 for texture features). Cases with discrepant segmentations (Dice coefficient <0.80) underwent consensus review by a third senior radiologist (>10 years’ experience). Segmentation quality was monitored through random 10% subset review throughout the study period.

**Figure 2:**
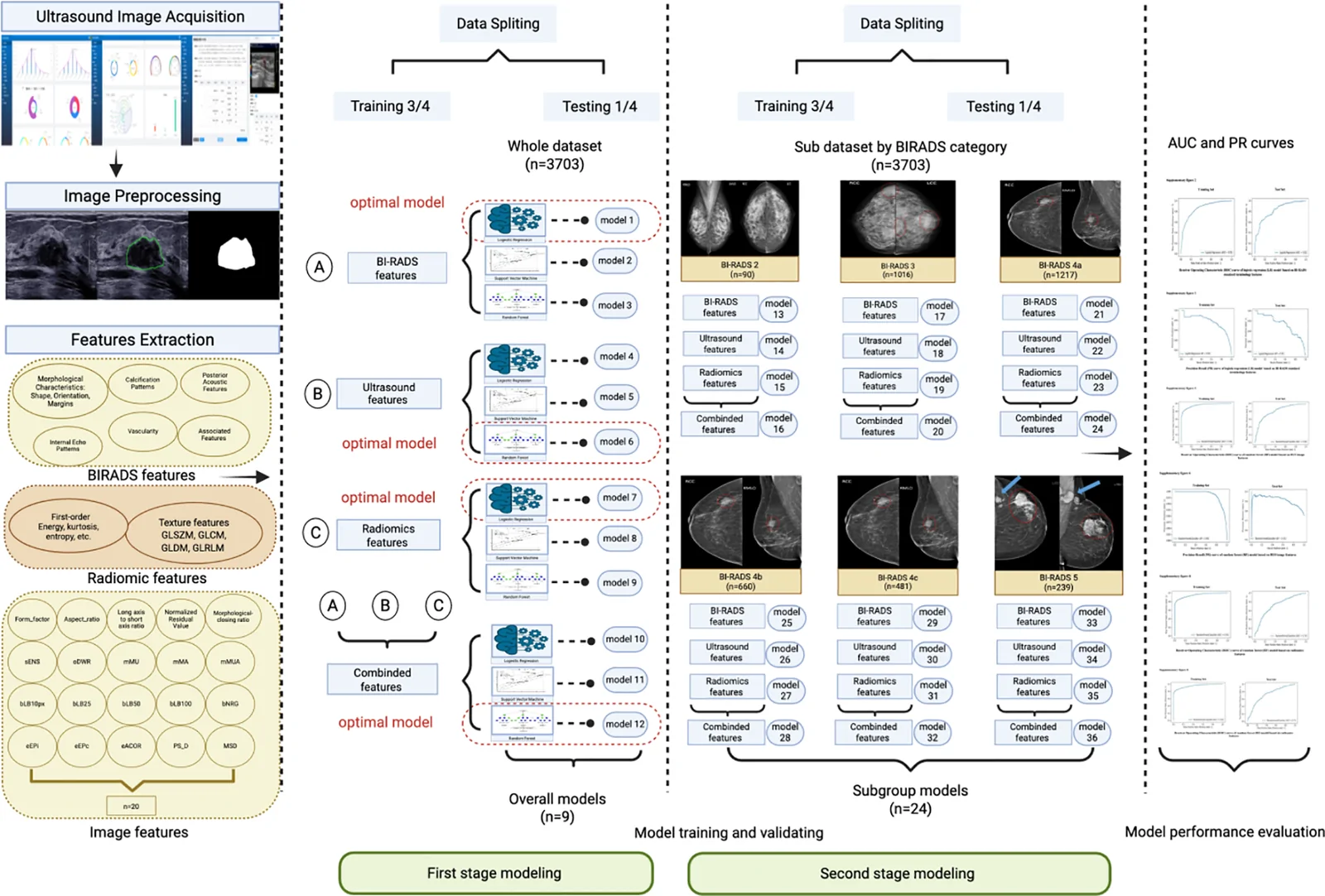
Process of model construction. The first column describes the extraction of image features, which are categorized into three types: BIRADS features, radiomic features, and image features. The second column involves modeling and validation for predicting the risk of malignant breast masses using these three types of features and their combined form via logistic regression, random forest, and support vector machine. The model within the red dashed box represents the optimal solution of the model under this feature set. The third column employs the three feature types and their combination to model and validate the prediction of BIRADS stratification for breast cancer malignancy using the optimal method-random forest. Model performance is evaluated by the area under the curve (AUC).

For radiomics, the following steps were followed, (i) Extracting radiomic features. The ultrasonic image features are extracted via Python. First-order statistical features: energy, kurtosis, entropy, etc.; morphological features, such as area, the maximum diameter of lesions, etc.; texture features, such as grey spatial region matrix (GLSZM), grey co-occurrence matrix (GLCM), grey dependency matrix (GLDM) and grey run length matrix (GLRLM), etc., as well as the above features were extracted after image transformation by filtering. (ii) Selection of radiomics features. We manually selected features. t-test was used to initially select characteristics, variables with *p* < 0.05 between the two groups were retained. Then the principal component analysis (PCA) was used to preserve the main components, explaining 90% of the differences in the original data, retaining components explaining 90% of variance (31 components). PCA was fitted on the training set only, with the transformation applied to both sets. LR, RF and SVM models were constructed based on the obtained BI-RADS standard terminology features, BUS image features, radiomics features, and a combination of these features. The process can be seen in **Figure 2**.

No missing values were present in the dataset. Cases with incomplete data were excluded during initial screening, resulting in complete-case analysis for all 3, 703 images. To address the imbalance of dataset, we applied Synthetic Minority Over-sampling Technique (SMOTE) to the training set, generating synthetic malignant samples to achieve approximately 1:1 class balance. Additionally, class weights inversely proportional to class frequencies were incorporated into all algorithms. Stratified sampling was used in cross-validation to maintain consistent class proportions. All features were standardized using Z-score normalization (z = (x-μ)/σ) based on training set parameters, which were then applied to the validation set to prevent data leakage.

To ensure unbiased model evaluation and prevent data leakage, we implemented a strict patient-level data partitioning strategy. The complete dataset of 2, 685 patients with 3, 703 ultrasound images was randomly divided into training and validation sets at the patient level using an 8:2 ratio with stratification by BI-RADS category and malignancy status. Training set (80%) comprised 2, 148 patients with 2, 979 images (2, 146 benign, 833 malignant). This set was used exclusively for model development, feature selection, and hyperparameter tuning via 5-fold stratified cross-validation. Each cross-validation fold maintained the patient-level separation to prevent images from the same patient appearing in both training and validation folds. Validation set (20%) comprised 537 patients with 724 images (616 benign, 108 malignant). This set was held out entirely during model development and used only for final performance evaluation. No information from the validation set influenced model selection or hyperparameter tuning. For patients with multiple ultrasound images (n=487 patients, 18.1% of cohort, contributing 1, 018 additional images), all images from the same patient were assigned exclusively to either the training set or the validation set. This patient-level split prevents data leakage that could occur if images from the same patient appeared in both partitions, which would artificially inflate performance estimates due to correlation between images from the same individual. The random split was performed using a fixed random seed (seed=42) to ensure reproducibility. Stratification ensured that the distribution of BI-RADS categories and malignancy ratios remained consistent between training and validation sets, as detailed in **Supplementary Table 3**.

### 2.7 Statistic analysis

The Python packages used were as follows: Python3.8 (https://www.python.org/); Pyradiomics3.0.1 (https://pyradiomics.readthedocs.io/en/latest/index.html); Pandas1.1.4 (https://pandas.pydata.org/pandas-docs/stable/index.html); Scikit-learn 0.23.2 (https://scikit-learn.org/stable/); Seaborn 0.11.0 (https://seaborn.pydata.org/index.html).

## 3 Results

### 3.1 Data description

Our study used a total of 3, 703 ultrasound images. These images were divided into a training set comprising 2, 979 images and a validation set comprising 724 images. Among these images, there were 2, 762 benign tumor images, and 941 malignant tumor images. The descriptive characteristics of these cohorts are detailed in **Table 1**. **Supplementary Table 4** presents the comparison of patient demographics and clinical characteristics. Detailed ICC values for each feature category are provided in **Supplementary Table 5**. These results confirm that the semi-automated segmentation approach produced robust and reproducible radiomics features suitable for model development.

**Table 1:** Baseline characteristics and comparison between training and validation sets.

Data set	Total number		Benign		Malignant		Benign vs. malignant	
	Patients	Images	Patients	Images	Patients	Images	Patients	Images
Training set	2, 148	2, 979	1, 653	2, 216	495	763	3.34:1	2.90:1
Test set	537	724	416	546	121	178	3.44:1	3.07:1
TOTAL	2, 685	3, 703	2, 069	2, 762	616	941	3.36:1	2.94:1

For patients with multiple images (n=487), all images were assigned to the same partition (training or validation) to ensure strict patient-level separation and prevent data leakage. No patient appeared in both training and validation sets.

### 3.2 Performance of models trained with whole dataset in malignant diagnosis

The performance of the three models, LR, SVM, and RF, was evaluated for breast cancer malignancy using different sets of features: BI-RADS standard terminology features, BUS image features, radiomics features, and combined features.

**Figure 3:**
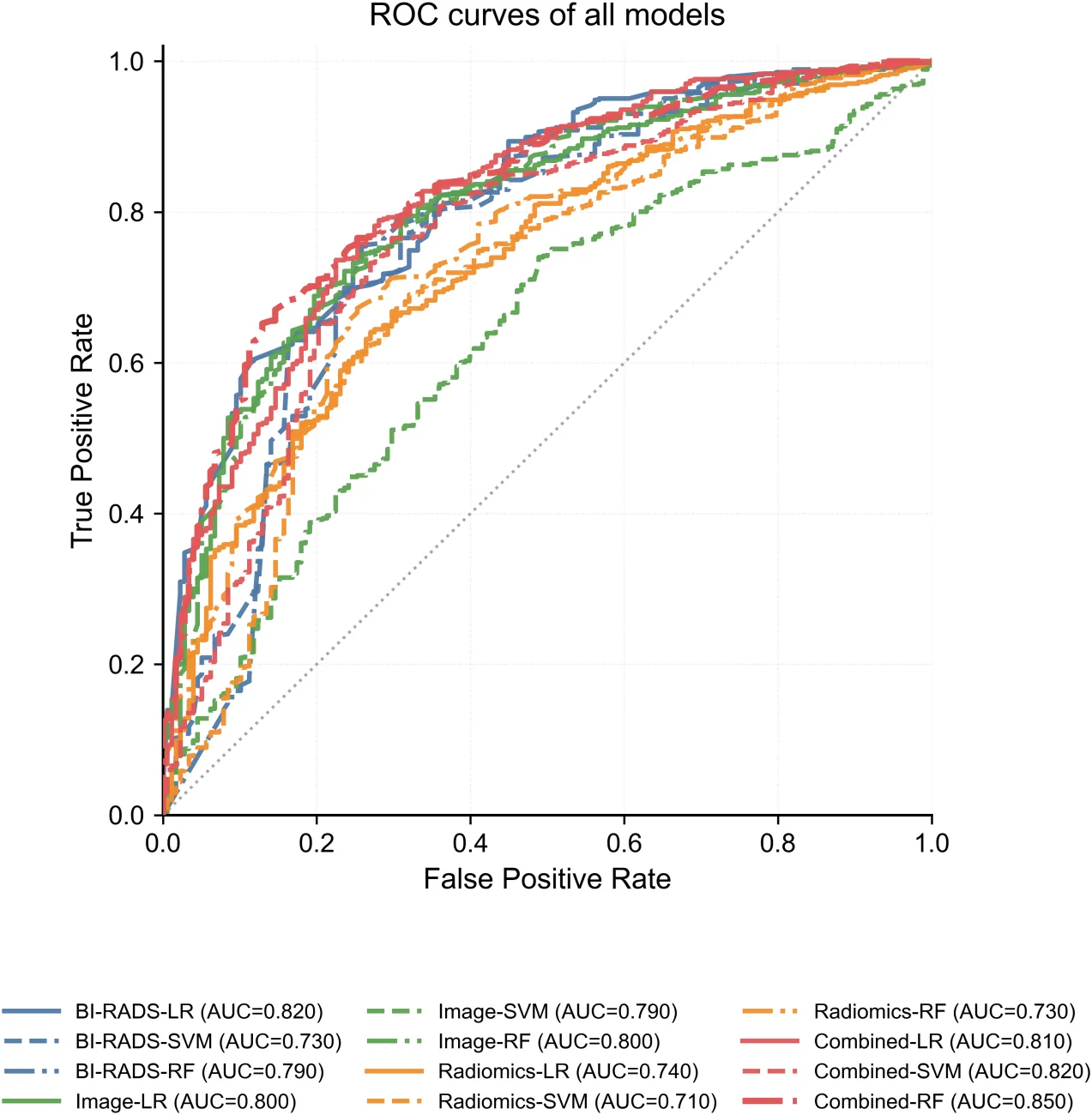
ROC curves of models trained with whole dataset.

**Figure 4:**
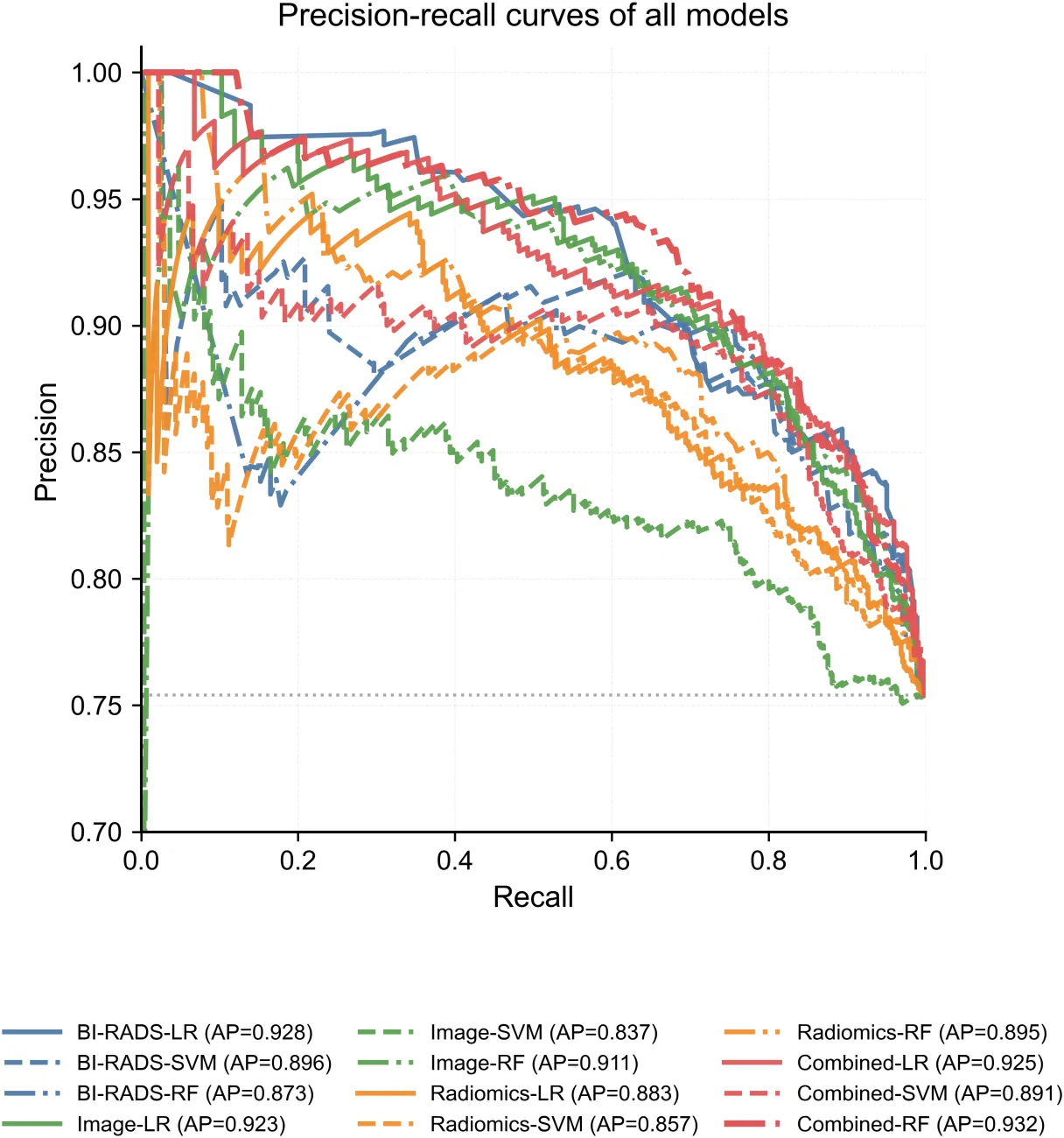
PR curves of models trained with whole dataset.

For BI-RADS standard terminology features, LR achieved the highest test set performance with an accuracy of 0.820 and an AUC of 0.820. SVM demonstrated slightly higher accuracy (0.830) but lower AUC (0.730), while RF showed moderate accuracy (0.800) and AUC (0.790). Using image features, all three models showed comparable accuracy performance on the test set: LR achieved 0.790, SVM reached 0.790, and RF obtained 0.790. However, RF and LR demonstrated high AUC (0.800), and followed by SVM (0.790). When using radiomics features, LR obtained the highest test set accuracy of 0.760 with an AUC of 0.740. SVM achieved slightly higher accuracy (0.770) but lower AUC (0.710), while RF showed moderate accuracy (0.760) and AUC (0.730). With combined features, RF achieved the highest overall performance with a test set accuracy of 0.820 and the best AUC of 0.850. SVM demonstrated identical accuracy (0.820) but lower AUC (0.820), while LR showed the lowest performance in this category with accuracy of 0.760 and AUC of 0.810. **Figure 3** shows the ROC curves for all models and **Figure 4** shows the PR curves for all models.

Across all feature sets, RF consistently demonstrated superior AUC performance, particularly with combined features (0.850) and competitive accuracy results. Considering that the RF model has more adjustable parameters and greater flexibility, combined with its superior AUC performance across most feature combinations, the RF model was considered as the best predictive performance model in our study. The detailed metrics on test sets are summarized in **Table 2**.

**Table 2:** The accuracy and AUC of the three models for overall BC benign/malignant diagnosis.

Original features	Model	TP	TN	FP	FN	Accuracy	Sensitivity	Specificity	AUC, 95% CI	F1 score
BI-RADS features	LR	489	94	84	57	0.805	0.896	0.528	0.820 (0.775, 0.856)	0.874
BI-RADS features	SVM	508	63	115	38	0.789	0.93	0.354	0.730 (0.671, 0.783)	0.869
BI-RADS features	RF	488	77	101	58	0.78	0.894	0.433	0.790 (0.735, 0.837)	0.860
Image features	LR	496	75	103	50	0.789	0.908	0.421	0.800 (0.765, 0.838)	0.866
Image features	SVM	543	1	177	3	0.751	0.995	0.006	0.790 (0.748, 0.830)	0.858
Image features	RF	499	83	95	47	0.804	0.914	0.466	0.800 (0.768, 0.839)	0.875
Radiomics features	LR	484	63	115	62	0.756	0.886	0.354	0.740 (0.706, 0.780)	0.845
Radiomics features	SVM	531	24	154	15	0.767	0.973	0.135	0.710 (0.655, 0.753)	0.863
Radiomics features	RF	492	55	123	54	0.756	0.901	0.309	0.730 (0.681, 0.769)	0.848
Combined features	LR	482	98	80	64	0.801	0.883	0.551	0.810 (0.811, 0.874)	0.870
Combined features	SVM	540	17	161	6	0.769	0.989	0.096	0.820 (0.786, 0.856)	0.866
Combined features	RF	504	79	99	42	0.805	0.923	0.444	0.850 (0.810, 0.875)	0.877

### 3.3 Performance of optimal models within each BI-RADS categories

Based on the optimal prediction models generated from the previous step, four overall benign-malignant classification models were constructed using BI-RADS standard terminology features, quantitative imaging features, radiomics features, and comprehensive multidimensional features as independent variables, respectively, with the pathological examination results of breast tumors (benign vs. malignant) as the dependent variable using the whole dataset. Their performance was assessed in each BI-RADS category.

**Table 3** summarizes the accuracy of each optimal model across different BI-RADS categories. (i) The optimal model using BI-RADS standard terminology features demonstrated perfect accuracy in BI-RADS 2 (1.000), excellent performance in BI-RADS 3 (0.951), good accuracy in BI-RADS 4a (0.878), and strong performance in BI-RADS 5 (0.906). However, performance declined in BI-RADS 4b (0.649) and BI-RADS 4c (0.536). (ii) The optimal model using image features achieved perfect accuracy in BI-RADS 2 (1.000), high performance in BI-RADS 3 (0.946), good accuracy in BI-RADS 4a (0.796), and moderate performance in BI-RADS 4b (0.723). Performance was limited in BI-RADS 4c (0.624) and BI-RADS 5 (0.674). (iii) The optimal model using radiomics features exhibited perfect accuracy in BI-RADS 2 (1.000), excellent performance in BI-RADS 3 (0.957), and good accuracy in BI-RADS 4a (0.842). Performance was moderate in BI-RADS 4b (0.664) but poor in BI-RADS 4c (0.495) and BI-RADS 5 (0.370). (iv) The optimal model using combined features achieved perfect accuracy in BI-RADS 2 (1.000), excellent performance in BI-RADS 3 (0.957), good accuracy in BI-RADS 4a (0.825), and moderate performance in BI-RADS 4b (0.701). Performance improved in BI-RADS 4c (0.634) and was excellent in BI-RADS 5 (0.870).

**Table 3:** The accuracy of the optimal models for BC malignant diagnosis in each BI-RADS categories (data from whole dataset).

Original features	BI-RADS category	Overall model	
		Training set	Test set
		(n=2, 979)	(n=724)
BIRADS features	2	0.983 (0.952, 1.000)	1.000 (1.000, 1.000)
	3	0.965 (0.951, 0.979)	0.951 (0.916, 0.987)
	4a	0.866 (0.841, 0.890)	0.878 (0.830, 0.926)
	4b	0.678 (0.631, 0.725)	0.649 (0.555, 0.744)
	4c	0.598 (0.535, 0.661)	0.536 (0.419, 0.654)
	5	0.814 (0.743, 0.884)	0.906 (0.805, 1.000)
Image features	2	1.000 (1.000, 1.000)	1.000 (1.000, 1.000)
	3	0.977 (0.967, 0.987)	0.946 (0.914, 0.979)
	4a	0.870 (0.849, 0.891)	0.796 (0.745, 0.847)
	4b	0.802 (0.768, 0.836)	0.723 (0.648, 0.800)
	4c	0.837 (0.800, 0.874)	0.624 (0.529, 0.718)
	5	0.839 (0.788, 0.891)	0.674 (0.538, 0.809)
Radiomics features	2	0.986 (0.960, 1.000)	1.000 (1.000, 1.000)
	3	0.972 (0.961, 0.983)	0.957 (0.928, 0.986)
	4a	0.864 (0.842, 0.885)	0.842 (0.795, 0.888)
	4b	0.728 (0.690, 0.766)	0.664 (0.584, 0.744)
	4c	0.668 (0.621, 0.716)	0.495 (0.398, 0.593)
	5	0.590 (0.521, 0.660)	0.370 (0.230, 0.509)
Combined features	2	0.986 (0.960, 1.000)	1.000 (1.000, 1.000)
	3	0.970 (0.958, 0.981)	0.957 (0.928, 0.986)
	4a	0.854 (0.831, 0.876)	0.825 (0.777, 0.873)
	4b	0.779 (0.744, 0.815)	0.701 (0.624, 0.779)
	4c	0.834 (0.797, 0.872)	0.634 (0.540, 0.728)
	5	0.881 (0.835, 0.927)	0.870 (0.772, 0.967)

Overall, the results indicate that the optimal models’ performance varies significantly depending on the BI-RADS category and the features used. All models consistently showed excellent performance in categories 2 and 3, with good accuracy in category 4a. Performance was generally moderate in category 4b, while category 4c presented the greatest challenge with variable results across different feature sets. Category 5 showed the most variable performance, ranging from poor (radiomics features) to excellent (combined features).

### 3.4 Performance of models trained with subgroup data in diagnosing BC within BI-RADS categories

According to the BI-RADS classification of breast masses, the data were divided into 6 datasets corresponding to BI-RADS categories 2, 3, 4a, 4b, 4c, and 5. Based on BI-RADS standard terminology features, quantitative imaging features, radiomics features, and comprehensive multidimensional features, benign-malignant classification models were constructed for each BI-RADS category, resulting in a total of 24 independent models. Model classification performance was evaluated using accuracy. Each case generated only one benign-malignant result, and for cases with multiple images, the output was determined based on the image with the highest degree of malignancy. In the training cohort (n=2, 979), the distribution was as follows: BI-RADS 2 comprised 74 cases (2.5%), BI-RADS 3 included 829 cases (27.8%), BI-RADS 4a represented the largest subset with 977 cases (32.8%), BI-RADS 4b contained 526 cases (17.7%), BI-RADS 4c included 380 cases (12.8%), and BI-RADS 5 comprised 193 cases (6.5%). The test set (n=724) demonstrated a similar distribution pattern: BI-RADS 2 with 16 cases (2.2%), BI-RADS 3 with 187 cases (25.8%), BI-RADS 4a with 240 cases (33.1%), BI-RADS 4b with 134 cases (18.5%), BI-RADS 4c with 101 cases (13.9%), and BI-RADS 5 with 46 cases (6.4%).

For BI-RADS standard terminology features, the optimal models achieved perfect accuracy in BI-RADS 2 (1.000), excellent performance in BI-RADS 3 (0.951), and good accuracy in BI-RADS 4a (0.867). Performance declined in higher-risk categories with BI-RADS 4b achieving 0.649, BI-RADS 4c reaching 0.551, but BI-RADS 5 demonstrating 0.813 accuracy. Using image features, models maintained perfect accuracy in BI-RADS 2 (1.000) and high performance in BI-RADS 3 (0.947). BI-RADS 4a achieved 0.800 accuracy, while BI-RADS 4b reached 0.709. The performance was more limited in BI-RADS 4c (0.604), but improved in BI-RADS 5 (0.848). For radiomics features, models demonstrated exceptional performance in lower-risk categories with perfect accuracy in BI-RADS 2 (1.000) and BI-RADS 3 (0.957). BI-RADS 4a achieved 0.825 accuracy, while BI-RADS 4b reached 0.679, BI-RADS 4c (0.584), but excellent in BI-RADS 5 (0.870). With combined features, models achieved perfect accuracy in BI-RADS 2 (1.000) and excellent performance in BI-RADS 3 (0.957). BI-RADS 4a demonstrated 0.804 accuracy, while BI-RADS 4b reached 0.694, BI-RADS 4c achieved 0.623. The highest-risk category BI-RADS 5 showed excellent performance with 0.870 accuracy. More details can be seen in **Table 4**.

**Table 4:** The accuracy of the optimal models for BC malignant diagnosis in each BI-RADS categories (data from each BI-RADS categories).

Original features	BI-RADS category	Subgroup model	
		Training set	Test set
BIRADS features	2	1.000 (1.000, 1.000)	1.000 (1.000, 1.000)
	3	0.965 (0.951, 0.979)	0.951 (0.916, 0.987)
	4a	0.863 (0.838, 0.888)	0.867 (0.817, 0.916)
	4b	0.683 (0.636, 0.731)	0.649 (0.555, 0.744)
	4c	0.653 (0.593, 0.715)	0.551 (0.433, 0.668)
	5	0.864 (0.803, 0.926)	0.813 (0.677, 0.948)
Image features	2	1.000 (1.000, 1.000)	1.000 (1.000, 1.000)
	3	0.999 (0.996, 1.000)	0.947 (0.914, 0.979)
	4a	0.868 (0.957, 0.979)	0.800 (0.749, 0.851)
	4b	0.876 (0.848, 0.905)	0.709 (0.632, 0.786)
	4c	0.866 (0.832, 0.900)	0.604 (0.509, 0.699)
	5	0.948 (0.917, 0.979)	0.848 (0.744, 0.952)
Radiomics features	2	1.000 (1.000, 1.000)	1.000 (1.000, 1.000)
	3	1.000 (1.000, 1.000)	0.957 (0.928, 0.986)
	4a	0.979 (0.969, 0.988)	0.825 (0.777, 0.873)
	4b	0.802 (0.768, 0.836)	0.679 (0.600, 0.758)
	4c	0.855 (0.820, 0.891)	0.584 (0.488, 0.680)
	5	0.990 (0.975, 1.000)	0.870 (0.772, 0.967)
Combined features	2	1.000 (1.000, 1.000)	1.000 (1.000, 1.000)
	3	0.999 (0.996, 1.000)	0.957 (0.928, 0.986)
	4a	0.961 (0.949, 0.973)	0.804 (0.754, 0.854)
	4b	0.791 (0.756, 0.826)	0.694 (0.616, 0.772)
	4c	0.837 (0.800, 0.874)	0.623 (0.529, 0.718)
	5	0.979 (0.959, 0.999)	0.870 (0.772, 0.967)

### 3.5 SHAP-based model interpretability analysis

To improve model transparency and clinical interpretability, SHapley Additive exPlanations (SHAP) analysis was performed for the best-performing combined-feature Random Forest model. TreeExplainer was used to calculate SHAP values in the validation cohort, with malignant prediction defined as the positive class. Global feature importance was quantified using the mean absolute SHAP value of each feature. A SHAP bar plot was generated to rank the most influential features according to their average absolute contribution to the model output.

SHAP analysis was performed to interpret the best-performing combined-feature Random Forest model. The SHAP bar plot identified A3, A1, C1, B1, D2, and D1 as the most influential BI-RADS-related descriptors contributing to malignant risk prediction. Quantitative ultrasound features, including sENS, form factor, oDWR, aspect ratio, Is, and eACOR, also contributed substantially to the model output. In addition, radiomics-derived features, including wavelet-HH_ngtdm_Coarseness, wavelet-LH_ngtdm_Strength, and original_gldm_DependenceNonUniformity, were among the top-ranked contributors. These findings indicate that the combined-feature Random Forest model integrated semantic BI-RADS descriptors, quantitative ultrasound image features, and radiomics-derived texture information for malignant risk stratification.

**Figure 5:**
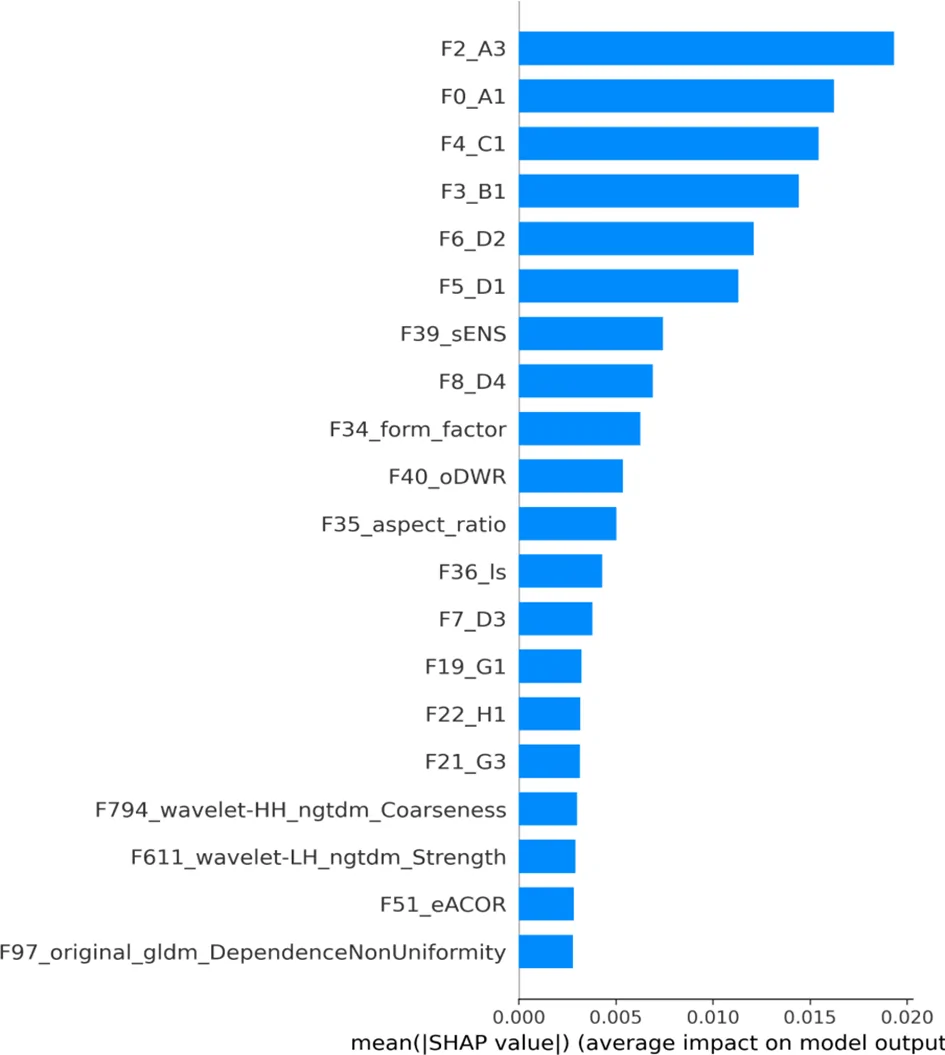
SHAP-based interpretability analysis of the combined-feature random forest model. The SHAP bar plot shows the top 20 features ranked by mean absolute SHAP value, reflecting their average contribution to malignant risk prediction. Higher mean absolute SHAP values indicate greater influence on model output. The top-ranked features included BI-RADS-related descriptors, quantitative ultrasound image features, and radiomics-derived texture features, indicating that the model incorporated complementary multimodal information for breast mass malignancy stratification. SHAP, SHapley Additive exPlanations; RF, Random Forest.

Overall, the SHAP results support the clinical plausibility of the multimodal fusion strategy. The highest-ranked predictors were not limited to high-dimensional radiomics features but also included interpretable BI-RADS terminology and quantitative morphological characteristics, suggesting that the model predictions were driven by clinically meaningful imaging information rather than purely opaque algorithmic patterns (see in **Figure 5**).

## 4 Discussion

### 4.1 Summary of key findings

In this retrospective cohort study, we developed and validated multimodal machine learning models integrating BI-RADS terminology features, ultrasound imaging characteristics, and radiomics features for breast mass malignancy risk stratification using data from 2, 685 patients with 3, 703 ultrasound images. The Random Forest model utilizing combined multimodal features achieved superior discriminative performance with an AUC of 0.850 (95% CI 0.810-0.875) compared to single-modality approaches. Model performance varied significantly across BI-RADS categories, demonstrating excellent accuracy in categories 2 (AUC 1.000) and 3 (AUC 0.947-0.957), acceptable performance in categories 5 (AUC 0.813-0.870) and 4a (AUC 0.800-0.867), but limited effectiveness in categories 4b (AUC 0.649-0.709) and 4c (AUC 0.551-0.623).

This represents the first comprehensive study to systematically evaluate machine learning integration of multiple ultrasound feature modalities across detailed BI-RADS subcategories, with subgroup-specific model training showing improved performance in certain categories, particularly BI-RADS 5. While previous studies have explored individual modalities or limited feature combinations in breast cancer diagnosis, none have comprehensively integrated BI-RADS terminology, quantitative imaging, and radiomics features with systematic evaluation across all BI-RADS subcategories using multiple machine learning approaches, nor have they provided detailed performance analysis for the challenging intermediate-risk categories 4a, 4b, and 4c that are critical for clinical decision-making.

### 4.2 Discussion over findings

Cancer is a highly complex disease involving a cascade of microscopic and macroscopic changes with mechanisms and interactions that are not yet fully understood (19). Medical image examination is the most effective method for diagnosis of BC, including digital mammogram, ultrasound, MRI, microscopic (histological) images, and infrared thermography (IRT) (20, 21). However, image interpretation is operator-dependent, which requires expertise. Indeed, studies demonstrated that poor-quality medical images evaluated by inexperienced radiologists can result in false positive radiological results and unnecessary biopsies of up to 50%. As is known, the pathological classification of BC remains a vital reference standard in the modality selection of treatment strategy, and it is usually determined according to pathologic results after complete surgical resection. However, despite the wide adoption of those invasive modalities, attempts to find alternative non-invasive methods never cease. Previous radiomics are mostly unimodal and based on image features of mammogram assessment, constraining AI methods to a single modality, significantly reducing clinical potential. Our results have implications for breast cancer screening and diagnostic workflows. Current breast ultrasound interpretation relies heavily on subjective BI-RADS assessments by radiologists, leading to high inter-observer variability and unnecessary biopsies in up to 50% of cases due to poor-quality images or inexperienced interpretation. Our multimodal machine learning approach produces diagnostic accuracy rates that compare favorably with published radiologist performance studies and addresses the clinical need for more objective, standardized breast mass evaluation. The Random Forest model using combined features achieved an AUC of 0.850, demonstrating superior performance compared to single-modality approaches and providing automated risk stratification that could support radiologists in challenging diagnostic scenarios.

Our model could integrate with and streamline radiologist workflow by providing objective probability assessments for breast mass malignancy across different BI-RADS categories. The model’s excellent performance in categories 2 (AUC 1.000) and 3 (AUC 0.947-0.957) could help confirm low-risk assessments, while acceptable performance in categories 4a (AUC 0.800-0.867) and 5 (AUC 0.813-0.870) provides additional diagnostic confidence for intermediate and high-risk lesions. This automated risk stratification could help radiologists focus their attention on the most challenging cases while reducing cognitive burden in routine evaluations. The integration of BI-RADS terminology, quantitative imaging features, and radiomics provides a comprehensive assessment that incorporates more information than traditional visual interpretation alone, potentially reducing the operator-dependency that has historically limited ultrasound diagnostic accuracy. The characteristics of clinical multimodal data provide the basis for the realization of accurate disease diagnosis. Different modalities of medical data provide patient diagnosis and treatment information from a specific perspective. Our study combined terminology features, image characteristics, and radiomics to generate models that successfully classify the subtypes of BC. To our knowledge, this is the first study with the most extensive dataset up to date to develop the machine learning algorithm that had a promising performance to automatically make the histologic subtype classification of breast cancer patients, which would dramatically help with the clinical efficiency and guide for physicians in decision making. In this study, we constructed LR, SVM, and RF models and found that RF was the optimal model (AUC 0.82) when trained by all data. Compared with the radiologists in the former study, the learning model had higher accuracy and better classification performance in predicting BC (22, 23).

What was pioneering for this research was that we proposed a machine-learning system with comprehensive coverage of almost all common histopathologic subtypes. And BIRADS 4 was further subclassified into finer divisions (4a, 4b, and 4c). The BI-RADS system has been widely used in breast cancer screening (24). There are seven categories in total (25). However, the BI-RADS system did not demonstrate the discrepancy among 4a, 4b, and 4c. This study puts forward the criteria for 4a, 4b, and 4c according to the number of suspicious malignant signs and the malignant risk ratio of signs (one suspicious sign is classified as 4a, two suspicious signs are judged as 4b, three suspicious signs are considered as 4c, and > 3 suspicious signs are regarded as 5 types). Our models are effective in discriminating BI-RADS 2, 3, and 5 sub-categories. We found that the accuracy of LR in BI-RADS 2, 3, and 5 is high, in BI-RADS 4a is medium, and in 4b and 4c is poor. We are the first study to define the differences between 4a, 4b, and 4c with the assistance of ML methods. Our findings are particularly relevant for the challenging BI-RADS category 4 subdivisions, where clinical decision-making is most complex. The model’s ability to distinguish between 4a, 4b, and 4c subcategories—defined by the number of suspicious malignant signs—represents a novel contribution to breast imaging informatics. While performance was limited in categories 4b (AUC 0.649-0.709) and 4c (AUC 0.551-0.623), these results highlight the inherent difficulty in these intermediate-risk categories and underscore the need for continued model refinement. The superior performance with combined multimodal features compared to single-modality approaches validates the clinical intuition that comprehensive feature integration improves diagnostic accuracy.

Radiogenomic aggregate data from radiology and genomics with the hypothesis that radiomic features are able to reflect macroscopic and molecular properties of tissues, thus could capture features from a full 3D volume of the tumor, avoiding sampling errors due to intra-tumor heterogeneity. Radiomics is a novel computer-aided technology, and has been increasingly gaining ground as a tool to maximize the information extracted from virtually any medical imaging modality, including MRI and mammograms, offers opportunity to improve sensitivity and specificity in diagnosis, prognosis, prediction, monitoring, image-based intervention, and assessment of therapeutic response (26). Radiomics showed huge potential in distinguish between malignant and benign lesions, assess the tumor subtype and its grade, assess the molecular expressions, and predict response to therapy and the risk of recurrence (27–30). In this study, 837 ultrasonic features were extracted using the open-source tool PYRADIOMICS, and 600 statistically different characteristics were retained by T test, and 31 principal components were retained by principal component analysis. The accuracy of this model is acceptable in BI-RADS 2, 3 and 4a. But interestingly, in current study, the predictive performance of the model based on radiomics was inferior to the other classification models in identifying subtypes, however, it also has potential for clinical application since the AUC of radiomics trained models is better when compared with previous studies (30, 31). This improvement may be largely due to the training sample sizes. However, the performance of models in BI-RADS 4 and 5 are relatively low, similar to the other 3 models, which, in results, suggested the urgent need for a publicly shared database to enable construction of more accurate artificial intelligence models.

The SHAP analysis improved the interpretability of the proposed multimodal model by identifying the features that contributed most strongly to malignant risk prediction. This is particularly important for AI-assisted medical decision support systems, in which transparency and clinical plausibility are essential for radiologist acceptance. The contribution of both BI-RADS-related descriptors and quantitative imaging/radiomics features supports the rationale for multimodal feature fusion and provides an interpretable basis for the performance of the combined-feature Random Forest model.

### 4.3 Limitations

Our study has several limitations. First, all data were derived from a single medical center, which may limit generalizability to different patient populations, imaging equipment, and radiologist interpretation practices; future studies should externally validate across multiple institutions with diverse patient demographics and imaging protocols. Second, the retrospective design and reliance on existing pathological diagnoses as ground truth may introduce spectrum bias, as cases with uncertain or borderline pathology might have been excluded from the original clinical dataset. Third, the model focused on diagnostic classification rather than prognostic assessment, limiting its utility for treatment planning and long-term patient management; future work should incorporate survival outcomes and treatment response data. Fourth, the relatively small sample sizes in certain BI-RADS subcategories, particularly categories 2 (n=90) and 5 (n=239), may have limited statistical power to detect subtle performance differences and could affect model stability in these subgroups. Fifth, the manual ROI selection process, while reducing inter-rater variability through semi-automated approaches, still introduces potential selection bias and may not fully represent the automated workflow that would be required for clinical implementation. Sixth, given the complexity of breast cancer heterogeneity, we did not examine model performance across different histological subtypes or molecular characteristics, which could provide more clinically relevant risk stratification for personalized treatment decisions.

Moreover, we acknowledge that deep learning models, particularly convolutional neural networks (CNNs), represent a contemporary approach in medical image analysis and have shown promising results in breast ultrasound classification. However, our study focused on traditional machine learning with radiomics features for several reasons: (i) Interpretability: Traditional ML models with radiomics features provide explicit feature importance rankings and SHAP values, enabling clinicians to understand which image characteristics drive predictions—a critical requirement for clinical acceptance and regulatory approval. (ii) Sample Size: Our dataset (2, 979 training images) is relatively small for training deep CNNs from scratch, which typically require tens of thousands of images to avoid overfitting. While transfer learning could mitigate this limitation, pre-trained models on natural images may not optimally capture ultrasound-specific patterns. (iii) Computational Efficiency: Radiomics-based models require significantly less computational resources for training and deployment compared to deep learning, making them more accessible for resource-limited clinical settings. (iv) Clinical Integration: Radiomics features align with BI-RADS descriptors familiar to radiologists, facilitating integration into existing clinical workflows. Future studies should directly compare radiomics-based and deep learning approaches on larger, multi-center datasets to determine optimal strategies for different clinical contexts.

## 5 Conclusions

This study demonstrates that multimodal machine learning models integrating BI-RADS terminology features, ultrasound imaging characteristics, and radiomics can effectively stratify breast mass malignancy risk, with the Random Forest model using combined features achieving superior discriminative performance compared to single-modality approaches. This represents a comprehensive study to systematically integrate multiple ultrasound feature modalities with machine learning across all BI-RADS subcategories, establishing a foundation for AI-assisted breast cancer diagnosis that could reduce unnecessary biopsies, improve diagnostic consistency, and support radiologist decision-making in routine clinical practice, though multicenter validation and model refinement for challenging intermediate-risk categories remain essential before widespread clinical implementation. While our results are promising, multicenter external validation is essential before clinical implementation to ensure model generalizability across diverse clinical settings and patient populations.

## Data Availability

The raw data supporting the conclusions of this article will be made available by the authors, without undue reservation.
