# Correction: Multimodal feature fusion model for breast mass malignant risk stratification

**DOI:** 10.3389/fonc.2026.1936161

**Published:** 2026-08-17

**Authors:** Shengxin Pei, Xiumei Tang, Hongxia Su, Jingyan Liu, Zihan Lan, Siyu Wang, Yulan Peng

**Affiliations:** 1 Department of Ultrasound, West China Hospital/West China School of Medicine, Sichuan University, Chengdu, China; 2 Department of Ultrasound Medicine, The First Hospital of Northwest University, Xi’an, Shaanxi, China; 3 Institute of Hospital Management, West China Hospital of Sichuan University, Chengdu, China; 4 State Key Laboratory of Respiratory Health and Multimorbidity, Chengdu, Sichuan, China; 5 West China School of Nursing, Sichuan University, Chengdu, China; 6 Department of Outpatient, West China Hospital of Sichuan University, Chengdu, China

**Keywords:** artificial intelligence, breast cancer, machine learning, multimodal data, risk stratification

An incorrect **Funding** statement was provided. The funder Qinghai University Medical Faculty education and teaching reform project (qyjg202221) to SP was erroneously included in the **Funding** statement. This funder has now been removed.

The correct **Funding** statement reads:

The author(s) declared that financial support was received for this work and/or its publication. This study was funded by Sichuan Science and Technology Program (Grant No. 2025ZDZX0012) to YP.

The original version of this article has been updated.

