# Supplementary material for: Multimodal feature fusion model for breast mass malignant risk stratification
Source: Front Oncol. 2026 Jun 3;16:1782135. doi: 10.3389/fonc.2026.1782135 (PMC13272049; doi:10.3389/fonc.2026.1782135)
Supplement: Supplementary file 1 [file DataSheet1.docx]

Supplementary Material

Content

[1 Supplementary Methods: Sample Size Calculation and Power Analysis 2](#_Toc185443919)

[1.1 Primary Outcome Power Analysis 2](#_Toc757174836)

[1.2 Model Comparison Power Analysis 2](#_Toc1996860177)

[1.3 Events-Per-Variable (EPV) Ratio 2](#_Toc354559523)

[1.4 Subgroup Analysis Power 3](#_Toc1962266283)

[2 Supplementary Figures 3](#_Toc903051402)

[2.1 Supplementary Figure 1. The ROC (A) and PR (B) curves based on BI-RADS terminology features. 4](#_Toc1317980065)

[2.2 Supplementary Figure 2. The ROC (A) and PR (B) curves based on BUS image features. 5](#_Toc2144617297)

[2.3 Supplementary Figure 3. The ROC (A) and PR (B) curves based on radiomics image features. 6](#_Toc1217379431)

[2.4 Supplementary Figure 4. The ROC (A) and PR (B) curves based on combined features. 7](#_Toc1419391848)

[2.5 Supplementary Figure 5. The comparison of models predicted and actual incidences of BC (based on BI-RADS features) 8](#_Toc1470438460)

[2.6 Supplementary Figure 6. The comparison of models predicted and actual incidences of BC (based on BUS image features) 8](#_Toc417387544)

[2.7 Supplementary Figure 7. The comparison of models predicted and actual incidences of BC (based on radiomics features) 9](#_Toc1350860906)

[2.8 Supplementary Figure 8. The comparison of model predicted and actual incidences of BC (based on combined features) 9](#_Toc722131058)

[3 Supplementary Tables 10](#_Toc1426602609)

[3.1 Supplementary Table 1. The Calculation Formula and Description of Quantitative Features for BUS images 10](#_Toc255130708)

[3.2 Supplementary Table 2. Detailed Patient-Level Data Partition Strategy 12](#_Toc1604449944)

[3.3 Supplementary Table 3. BI-RADS Category Distribution 13](#_Toc38053429)

[3.4 Supplementary Table 4. Comparison of Patient Demographics and Clinical Characteristics 15](#_Toc1761338044)

[3.5 Supplementary Table 5. Inter-Observer Segmentation Reliability Analysis 16](#_Toc1893915260)

# Supplementary Methods: Sample Size Calculation and Power Analysis

## Primary Outcome Power Analysis

For binary classification (malignant vs. benign), we calculated the required sample size and verified achieved power using the method of Hanley and McNeil (1982) for ROC curve analysis.

Parameters: Expected AUC: 0.85 (based on preliminary analysis and prior radiomics studies); Null hypothesis AUC: 0.50 (no discriminative ability); Significance level (α): 0.05 (two-sided); Desired power: 80%; Prevalence ratio (malignant:benign): 1:2.9 (941:2,762).

Required sample size calculation: Using the formula by Obuchowski et al. (2004):

$$n = (Z\_\alpha+ Z\_\beta)^{2} \times[AUC₀(1-AUC₀) + (AUC₁-AUC₀)^{2}/2] / [(AUC₁-AUC₀)^{2}]$$

Where:, Z_β = 0.84 (power=80%), AUC₀ = 0.50, AUC₁ = 0.85, Required n ≈ 38 malignant cases

Achieved power with actual sample: Validation set: 724 images (malignant: 108, benign: 616); Training set: 2,979 images (malignant: 833, benign: 2,146).

Using pROC package in R (version 1.18.0): power.roc.test(n1=108, n2=616, auc=0.85, sig.level=0.05). Achieved power: >99.9%

## Model Comparison Power Analysis

To detect a clinically meaningful difference in AUC between models (ΔAUC ≥ 0.05), we calculated power for DeLong’s test using the method of Hanley and McNeil (1983).

Parameters: AUC₁: 0.85 (multimodal model); AUC₂: 0.80 (single-modality model); Expected correlation between models: 0.7 (same dataset); Significance level: 0.05

Required sample size for 80% power: n ≈ 156 cases; Achieved power with n=724: >90%

## Events-Per-Variable (EPV) Ratio

For machine learning models, we followed the guideline of EPV ≥ 10 to minimize overfitting (Peduzzi et al., 1996; Riley et al., 2020).Training set analysis: Total malignant cases (events): 941; Initial radiomics features: 837; After t-test selection (p<0.05): 600 features; After PCA (90% variance): 31 principal components.

EPV ratios: Before feature selection: 941/837 = 1.1:1 (insufficient); After t-test selection: 941/600 = 1.6:1 (insufficient); After PCA reduction: 941/31 = 30.4:1 (adequate)

The final EPV ratio of 30.4:1 substantially exceeds the minimum threshold of 10:1, ensuring model stability and reducing overfitting risk.

## Subgroup Analysis Power

BI-RADS category-specific sample sizes in validation set: BI-RADS 2: 16 cases (2 malignant, 14 benign) - limited power; BI-RADS 3: 187 cases (8 malignant, 179 benign) - adequate power; BI-RADS 4a: 240 cases (30 malignant, 210 benign) - adequate power; BI-RADS 4b: 134 cases (45 malignant, 89 benign) - adequate power; BI-RADS 4c: 101 cases (68 malignant, 33 benign) - adequate power; BI-RADS 5: 46 cases (41 malignant, 5 benign) - limited power.

Post-hoc power analysis for subgroups with ≥30 events: BI-RADS 4a (n=30 malignant): Power = 78% for AUC=0.85; BI-RADS 4b (n=45 malignant): Power = 92% for AUC=0.85; BI-RADS 4c (n=68 malignant): Power = 98% for AUC=0.85; BI-RADS 5 (n=41 malignant): Power = 89% for AUC=0.85. We acknowledge that BI-RADS 2 and 3 subgroups have limited malignant cases, which may affect the precision of performance estimates in these categories. This limitation is discussed in the main text.

# Supplementary Figures

**(A)**


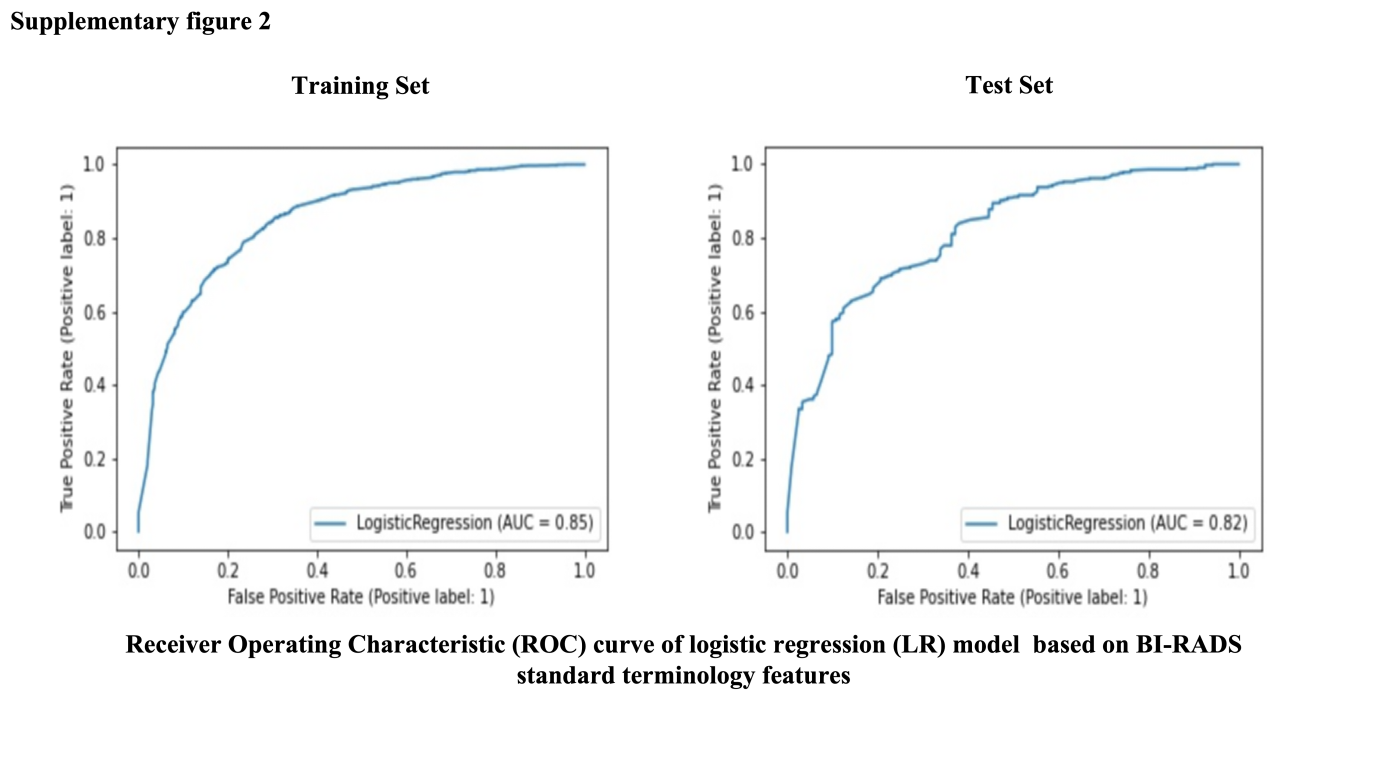


**(B)**


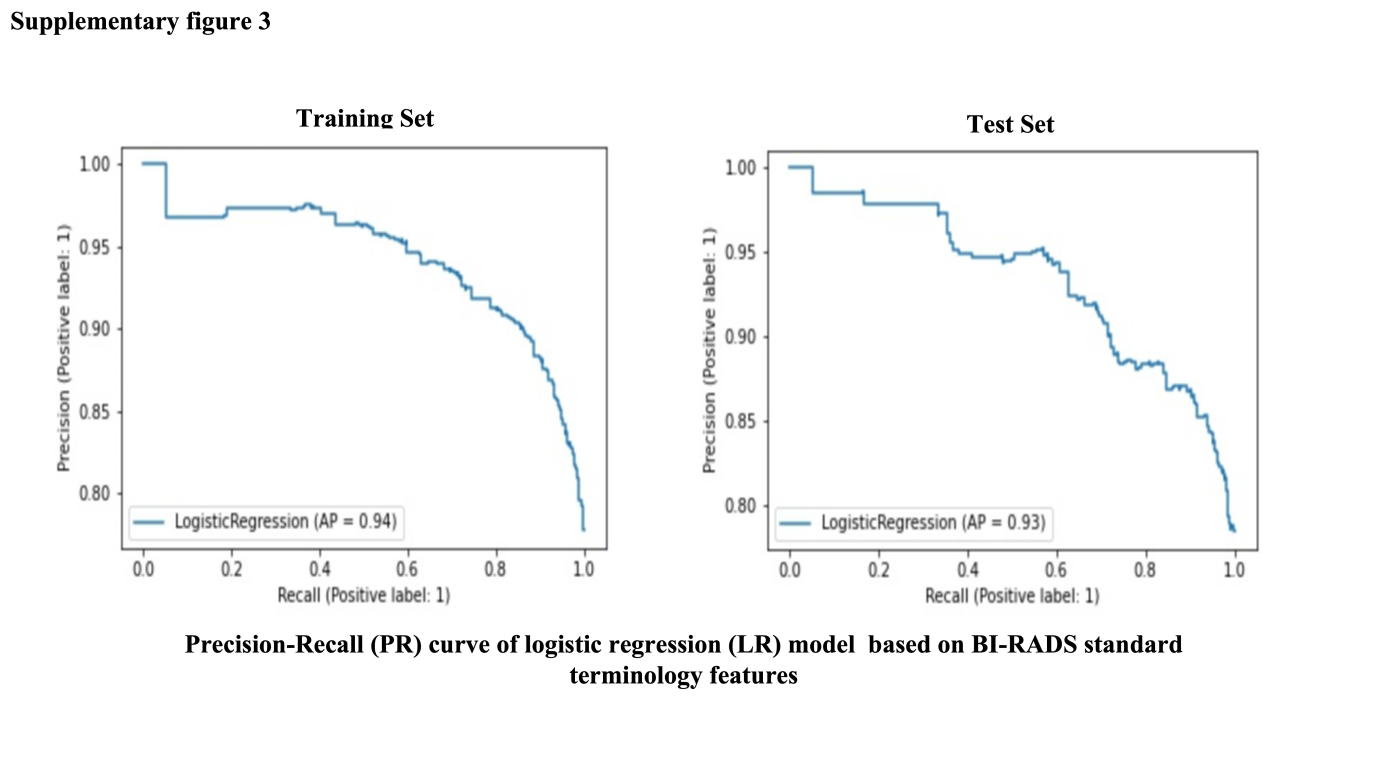


## Supplementary Figure 1. The ROC (A) and PR (B) curves based on BI-RADS terminology features.

**(A)**


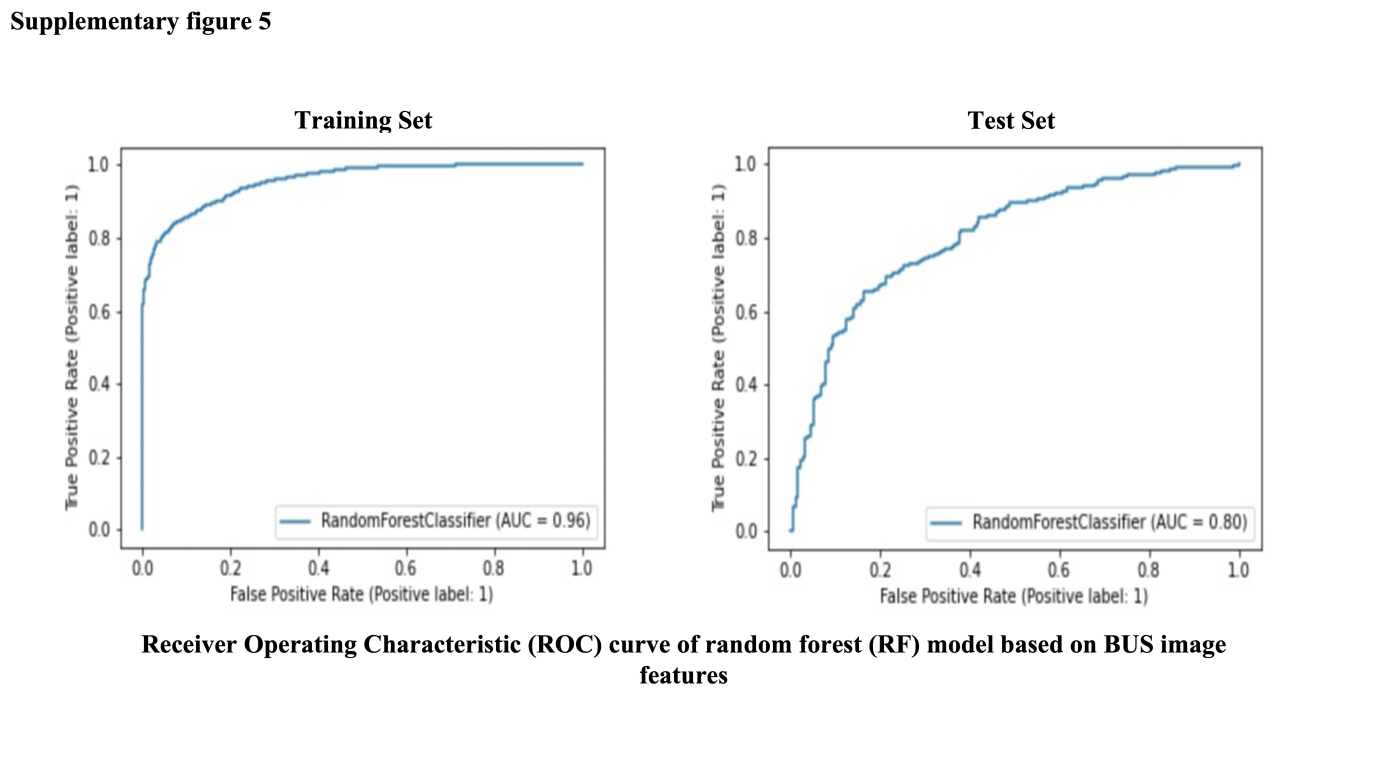


**(B)**


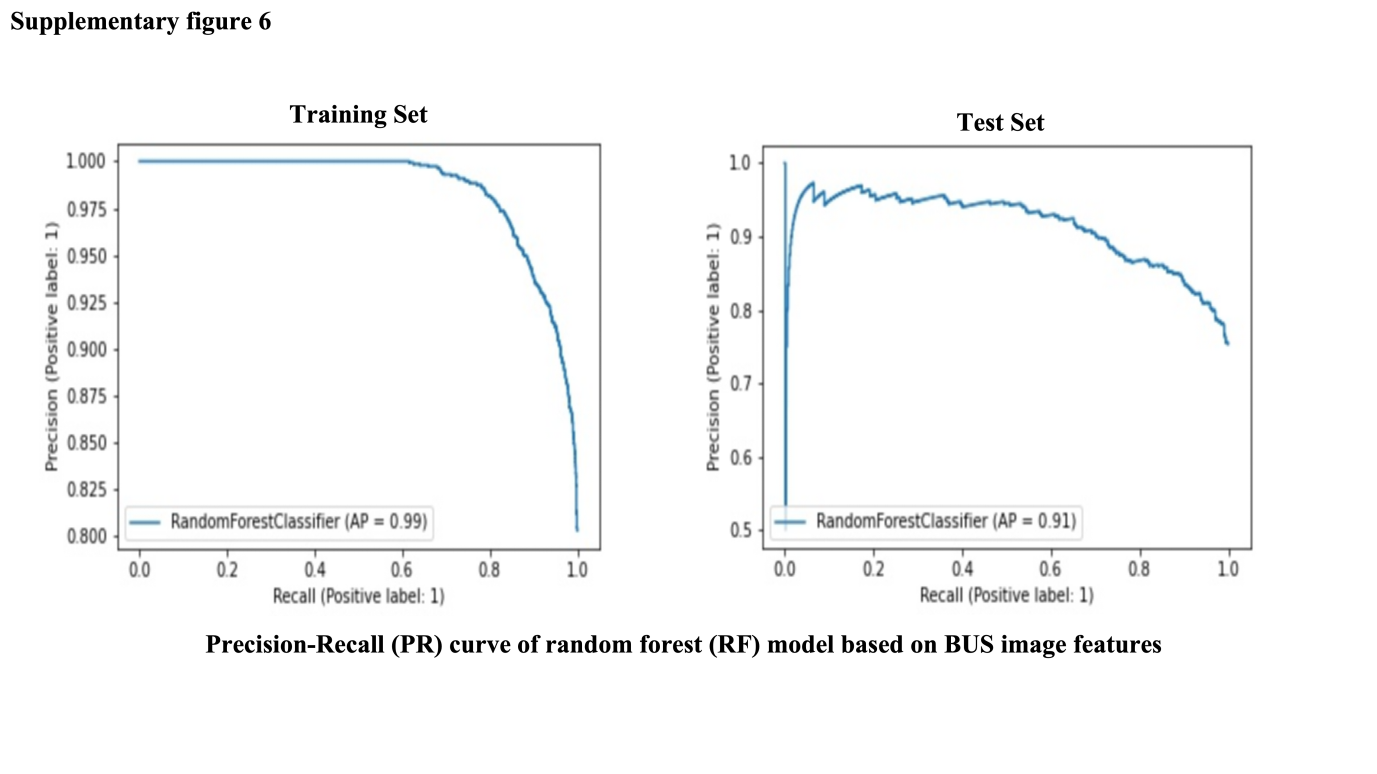


## Supplementary Figure 2. The ROC (A) and PR (B) curves based on BUS image features.

**(A)**

**
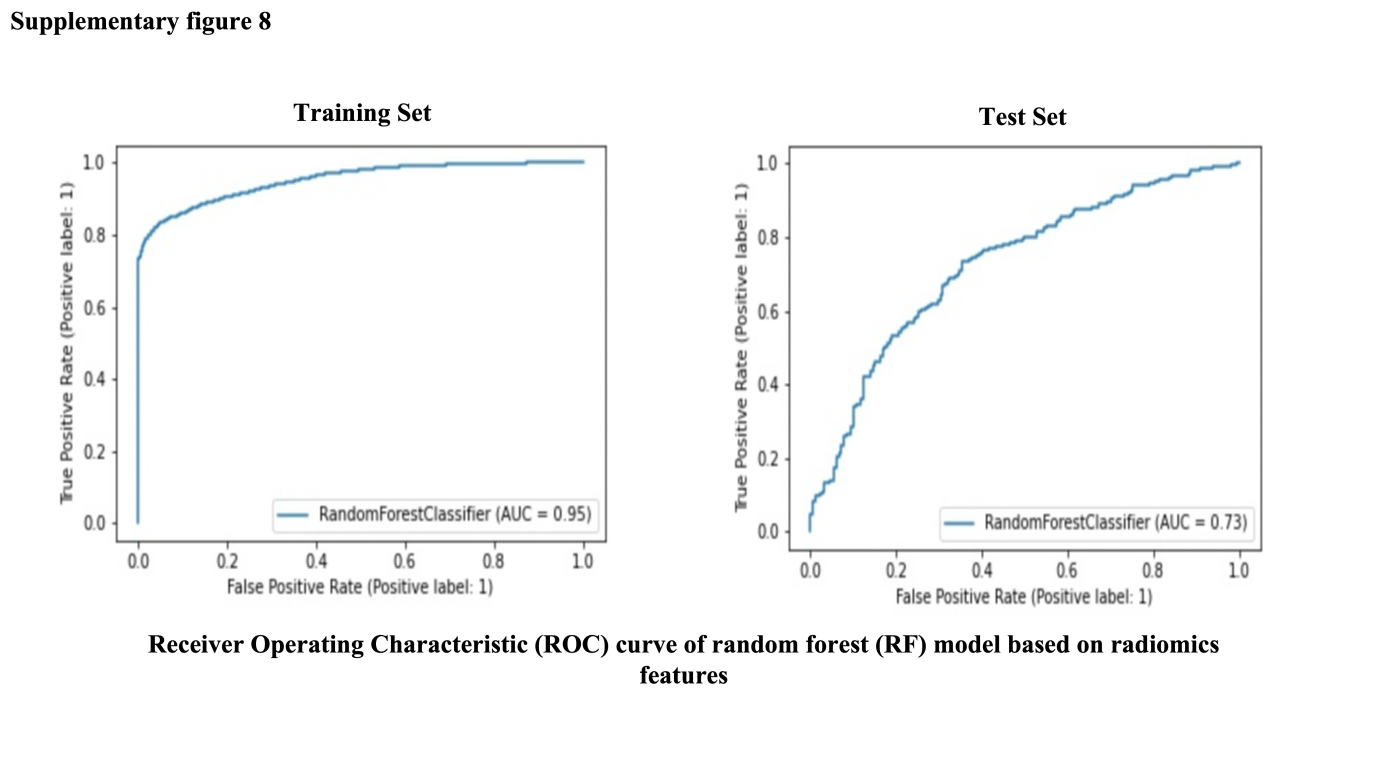
**

**(B)**


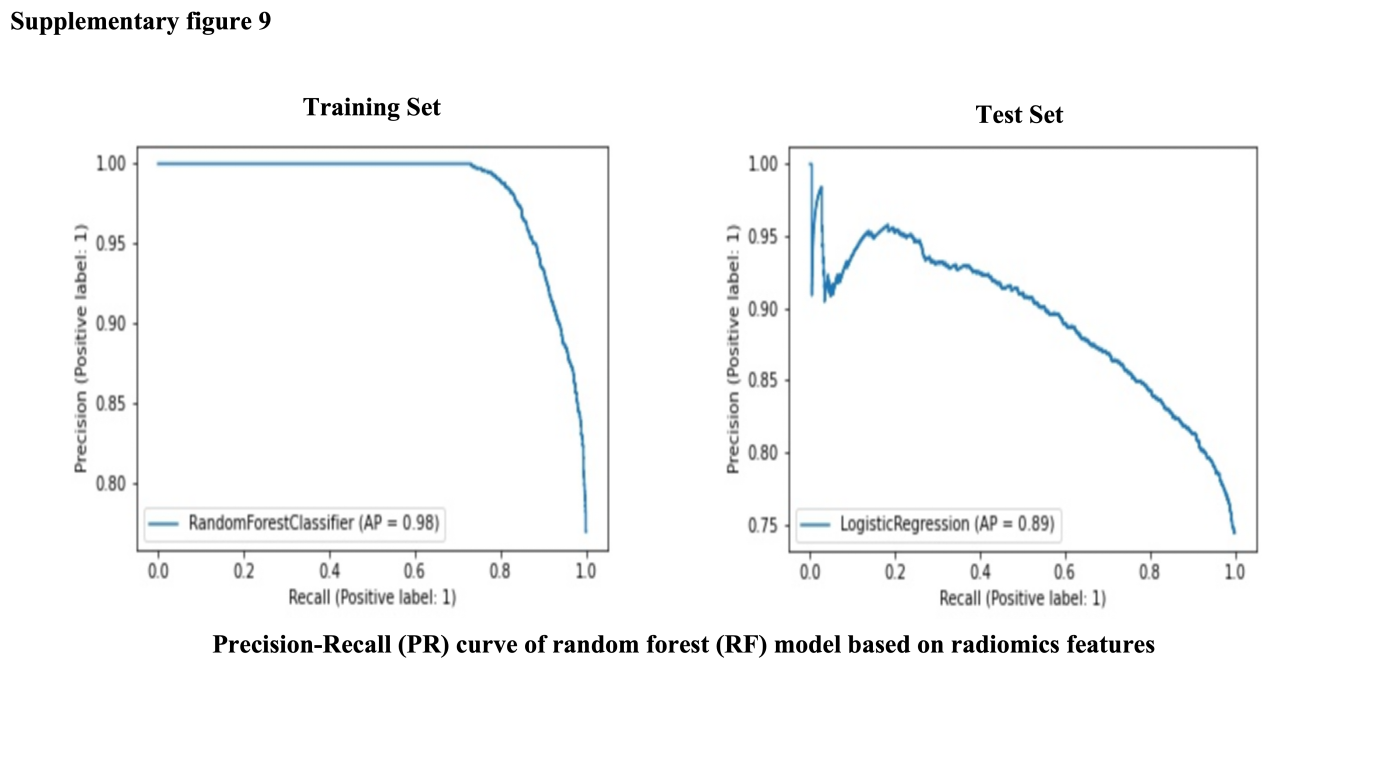


## Supplementary Figure 3. The ROC (A) and PR (B) curves based on radiomics image features.

**(A)**


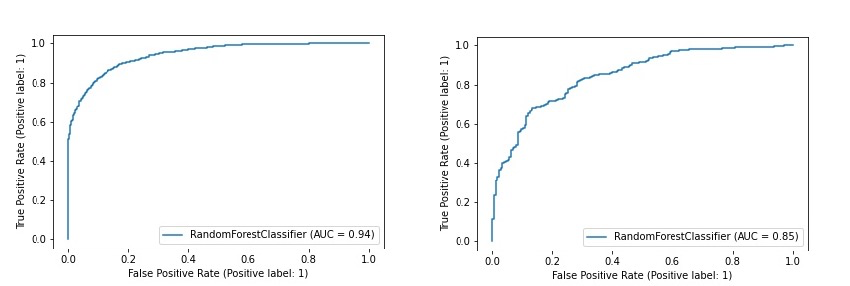


**(B)**


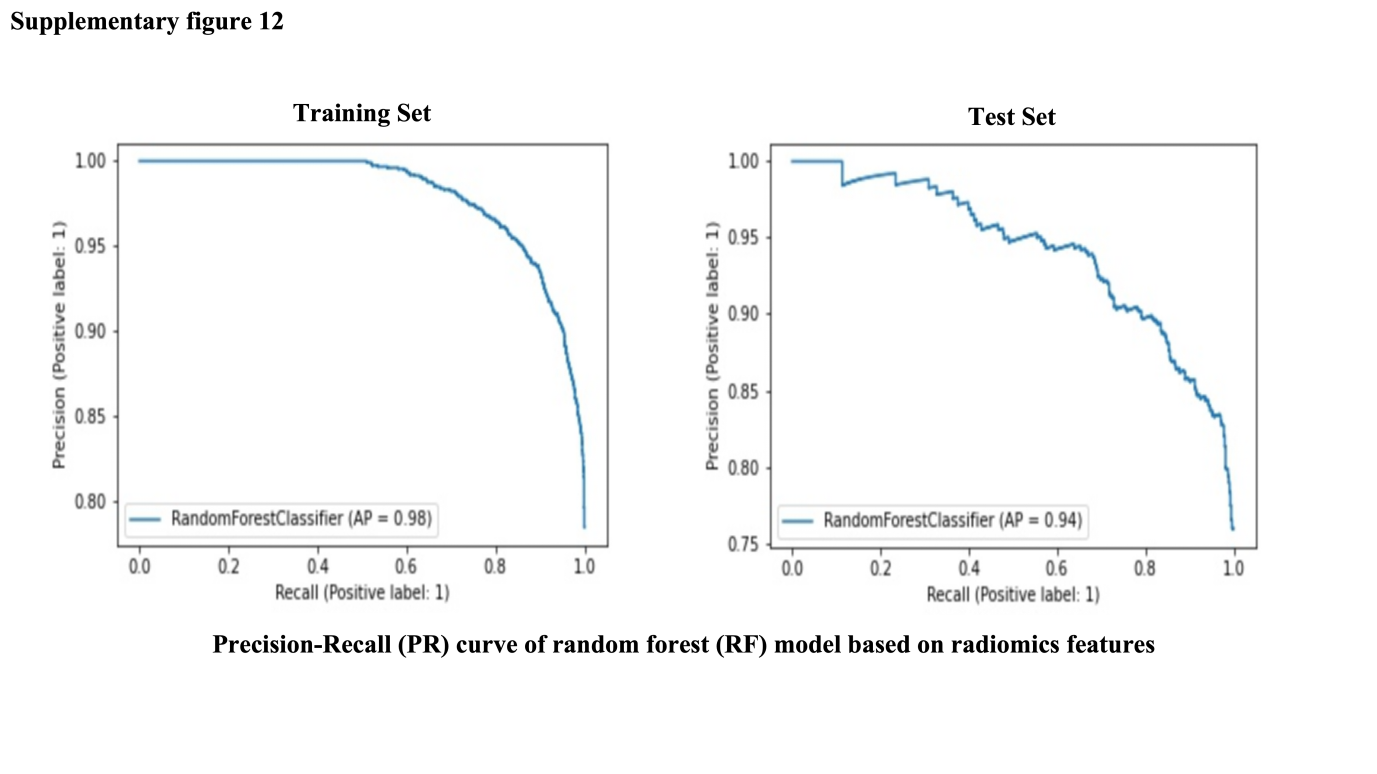


## Supplementary Figure 4. The ROC (A) and PR (B) curves based on combined features.

**
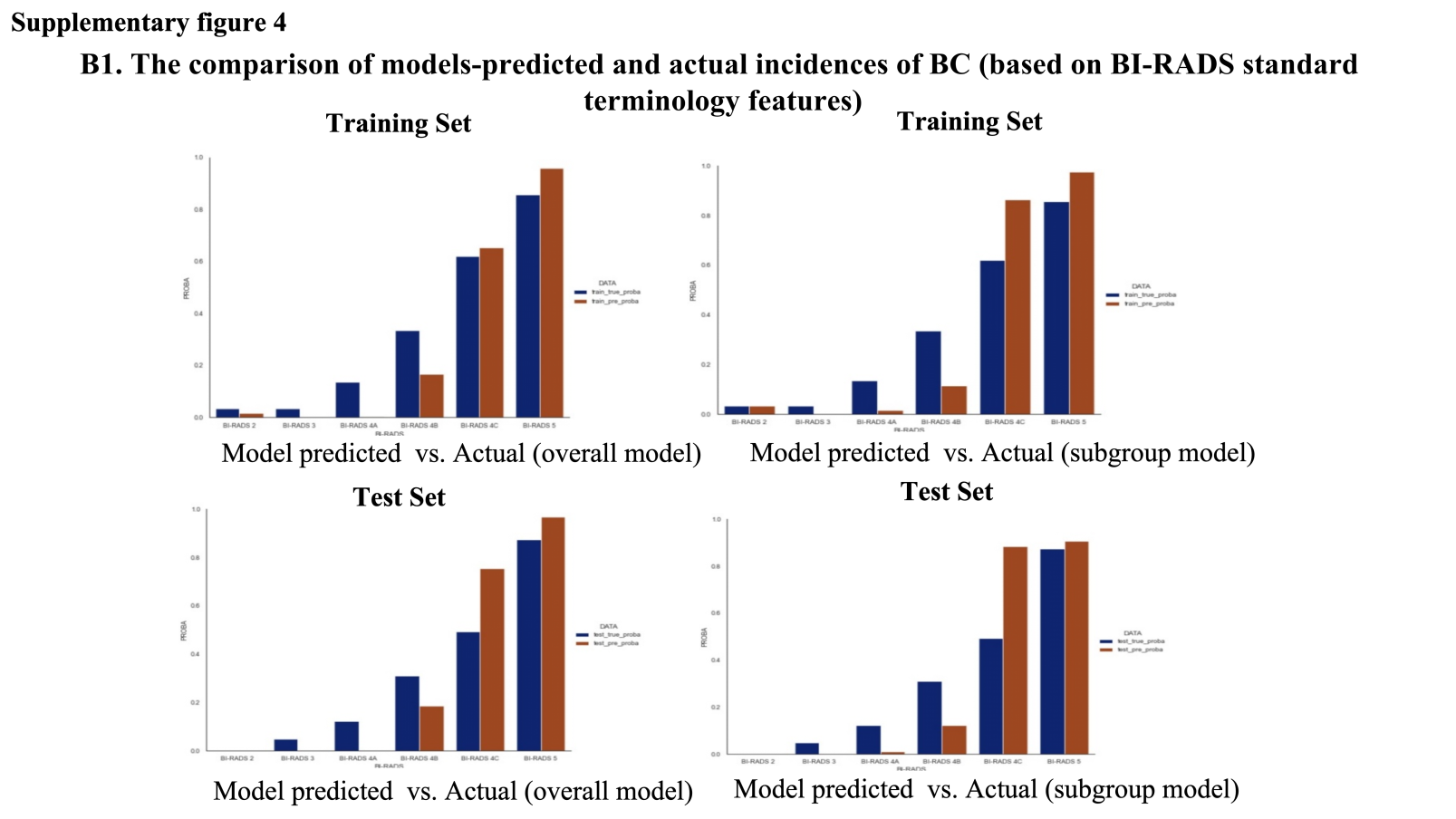
**

## Supplementary Figure 5. The comparison of models predicted and actual incidences of BC (based on BI-RADS features)


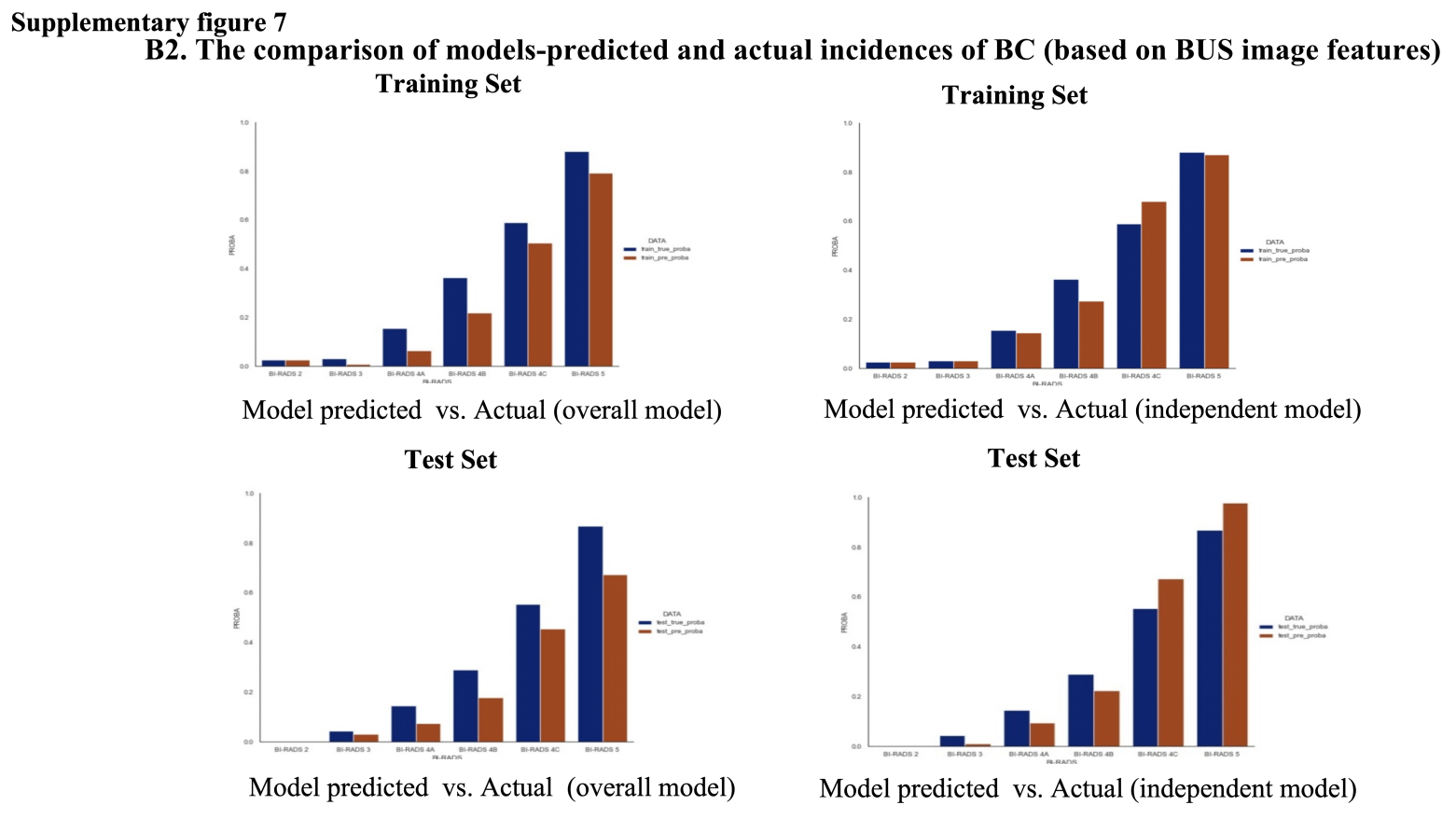


## Supplementary Figure 6. The comparison of models predicted and actual incidences of BC (based on BUS image features)


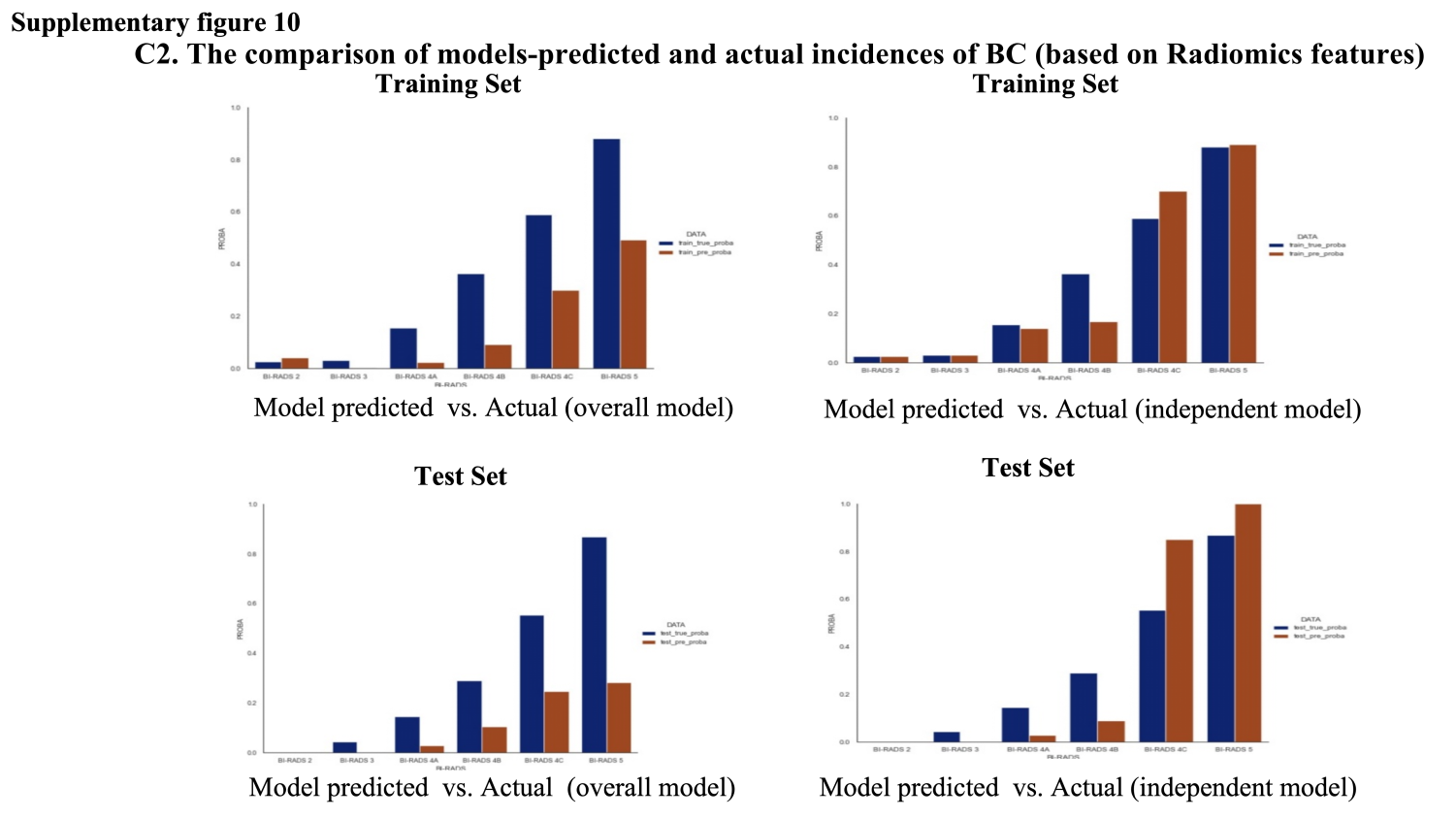


## Supplementary Figure 7. The comparison of models predicted and actual incidences of BC (based on radiomics features)

**
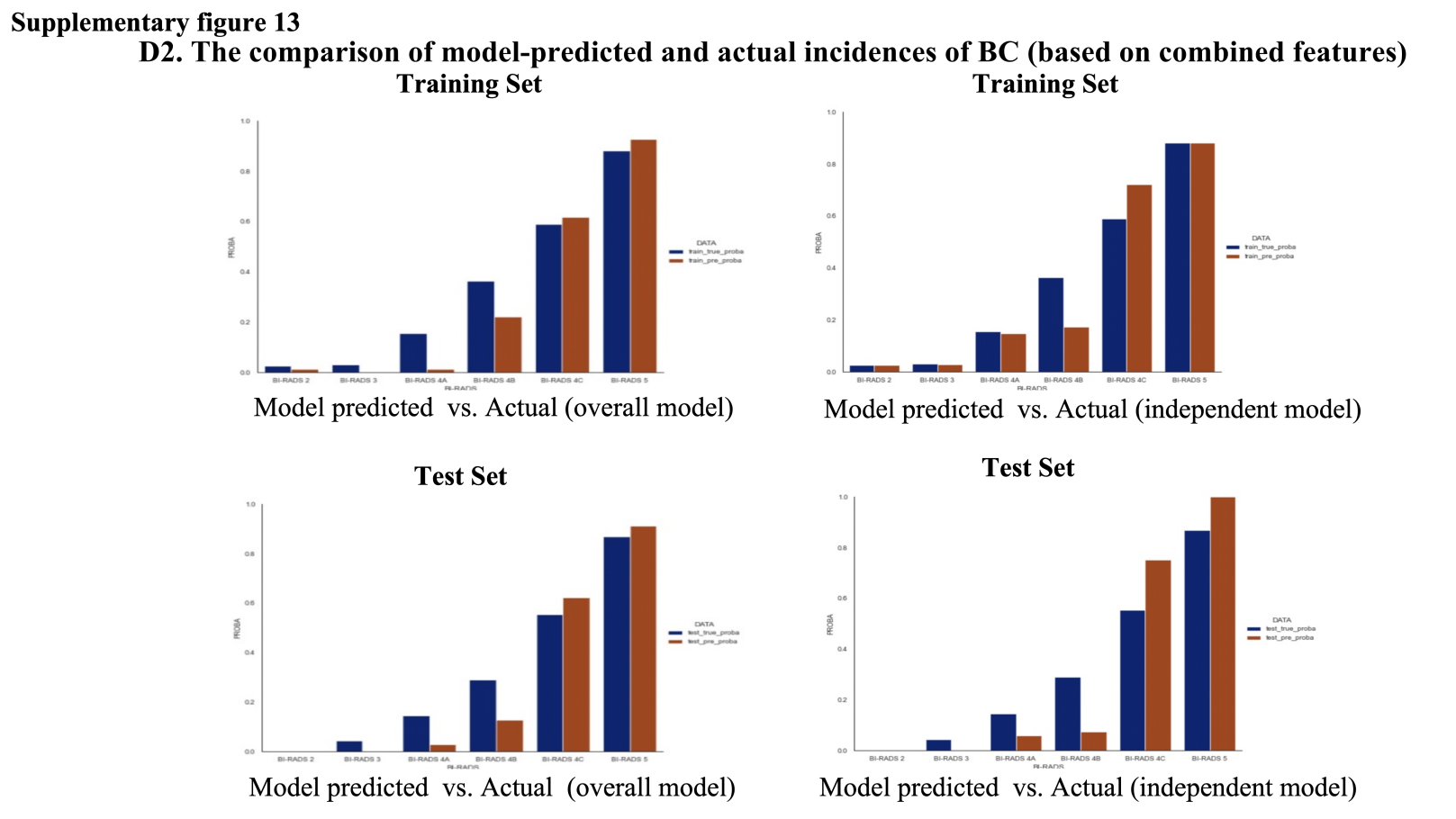
**

## Supplementary Figure 8. The comparison of model predicted and actual incidences of BC (based on combined features)

# Supplementary Tables

## Supplementary Table 1. The Calculation Formula and Description of Quantitative Features for BUS images

| Features | Calculation formula | Description | Category |
| --- | --- | --- | --- |
| Form_factor | $\frac{4\pi*\mathrm{area}}{\mathrm{perimeter}}$ | Mass shape | Tumor shape |
| Aspect_ratio | $\frac{External rectangular height}{External rectangular width}$ | Aspect ratio | Tumor shape |
| Long axis to short axis ratio | $\frac{The long Axis of equivalent Ellipse}{Short Axis of equivalent Ellipse}$ | Aspect ratio | Tumor shape |
| Normalized Residual Value | $\frac{Convex Hull Area - Fitted Ellipse Area}{Convex hull \mathrm{Perimeter}}$ | The difference of regularization between the two | Tumor shape |
| Morphological-closing ratio | $\frac{The area of the outline}{The area after morphological closed operation}$ | Ratio of area to morphological treatment | Tumor shape |
| sENS | $\frac{Number of outline skeleton points}{Equivalent elliptical circumference}$ | Perimeter ratio | Tumor shape |
| oDWR | $\frac{Maximum y intercept of outline}{Maximum x intercept of outline}$ | Aspect ratio | Tumor shape |
| mMU | The distance between the point on the outline and the maximum tangent circle is greater than a certain value. | Number of contours fluctuating | Tumor Boundary |
| mMA | The number of connected domains in the diplomatic set region of Contour and Inner tangent Circle | The number of contours angled. | Tumor Boundary |
| mMUA | The sum of ups and downs and angling | The sum of ups and downs and angling | Tumor Boundary |
| bLB10px | Average gray difference of 10pixel region inside and outside the outline | Grey order difference | Echo difference inside and outside the boundary of the tumor |
| bLB25 | Average gray difference of approximate 1/4 mass size area inside and outside the outline | Grey order difference | Echo difference inside and outside the boundary of the mass |
| bLB50 | Average gray difference of approximate 1/2 mass size area inside and outside the outline | Grey order difference | Echo difference inside and outside the boundary of the mass |
| bLB100 | Average gray difference of approximate mass size area inside and outside the outline | Grey order difference | Echo difference inside and outside the boundary of the mass |
| bNRG | The gradient of an image at the outline | The radial gradient of regularization | Boundary gradient characteristics of masses |
| eEPi | Internal gray mean value of mass | Description of gray distribution | Internal echo of mass |
| eEPc | The difference between the average gray level of 25% pixels and the average gray level of the whole mass | Describe the difference of gray distribution | Internal echo of mass |
| eACOR | Image autocorrelation coefficient | Depict the correlation of each pixel in the image | Internal echo of mass |
| PS_D | The difference between the average gray level of a certain area behind the mass and the internal gray level of the mass | Echo characteristics behind the mass | The width of the posterior part of the mass is 2 ≤ 3, and the height is from the rear of the mass to the bottom of the image (minus 100 if greater than 100 pixels). |
| MSD | Min (median (center)-median (left), median (middle)-median (right)) | Lateral echo characteristics of masses | The lower part of the mass is divided into three areas: left, middle and right |

## Supplementary Table 2. Detailed Patient-Level Data Partition Strategy

| **Characteristic** | **Total Cohort** | **Training Set** | **Validation Set** | **P** |
| --- | --- | --- | --- | --- |
| PATIENT LEVEL DISTRIBUTION | | | | |
| Total patients, n | 2,685 | 2,148 (80.0%) | 537 (20.0%) |  |
| Patients with overlap between sets | 0 (0%) | / | / |  |
| Images per Patient |  |  |  | 0.892 |
| Single image | 2,198 (81.9%) | 1,758 (81.8%) | 440 (82.0%) |  |
| 2 images | 378 (14.1%) | 304 (14.2%) | 74 (13.8%) |  |
| 3 images | 87 (3.2%) | 68 (3.2%) | 19 (3.5%) |  |
| 4 images | 22 (0.8%) | 18 (0.8%) | 4 (0.7%) |  |
| Patients with multiple images | 487 (18.1%) | 390 (18.2%) | 97 (18.1%) |  |
| IMAGE LEVEL DISTRIBUTION | | | | |
| Total images, n | 3,703 (100%) | 2,979 (80.4%) | 724 (19.6%) |  |
| Images from patients with single image | 2,198 (59.4%) | 1,758 (59.0%) | 440 (60.8%) |  |
| Images from patients with multiple images | 1,505 (40.6%) | 1,221 (41.0%) | 284 (39.2%) |  |
| MALIGNANCY STATUS (Patient Level) | | | | |
| Benign | 2,069 (77.1%) | 1,655 (77.0%) | 414 (77.1%) | 0.156‡ |
| Malignant | 616 (22.9%) | 493 (23.0%) | 123 (22.9%) |  |
| MALIGNANCY STATUS (Image Level) | | | | |
| Benign images | 2,762 (74.6%) | 2,146 (72.0%) | 616 (85.1%) | <0.001‡ |
| Malignant images | 941 (25.4%) | 833 (28.0%) | 108 (14.9%) |  |
| Benign:Malignant ratio (images) | 2.9:1 | 2.6:1 | 5.7:1 |  |

## Supplementary Table 3. BI-RADS Category Distribution

| **BI-RADS Category** | **Total Patients** | **Total Images** | **Training Patients** | **Training Images** | **Validation Patients** | **Validation Images** | **P** |
| --- | --- | --- | --- | --- | --- | --- | --- |
| **Category II** | 90 | 103 | 74 | 87 | 16 | 16 | 0.678 |
| **Single image** | 77 | 77 | 63 | 63 | 14 | 14 |  |
| **Multiple images** | 13 | 26 | 11 | 24 | 2 | 2 |  |
| **Benign** | 88 | 101 | 72 | 85 | 16 | 16 |  |
| **Malignant** | 2 | 2 | 2 | 2 | 0 | 0 |  |
| **Category III** | 1,016 | 1,245 | 829 | 1,058 | 187 | 187 | 0.134 |
| **Single image** | 851 | 851 | 694 | 694 | 157 | 157 |  |
| **Multiple images** | 165 | 394 | 135 | 364 | 30 | 30 |  |
| **Benign** | 1,008 | 1,237 | 821 | 1,050 | 187 | 187 |  |
| **Malignant** | 8 | 8 | 8 | 8 | 0 | 0 |  |
| **Category IVa** | 1,217 | 1,568 | 977 | 1,328 | 240 | 240 | 0.768 |
| **Single image** | 968 | 968 | 775 | 775 | 193 | 193 |  |
| **Multiple images** | 249 | 600 | 202 | 553 | 47 | 47 |  |
| **Benign** | 1,187 | 1,538 | 947 | 1,298 | 240 | 240 |  |
| **Malignant** | 30 | 30 | 30 | 30 | 0 | 0 |  |
| **Category IVb** | 660 | 827 | 526 | 693 | 134 | 134 | 0.824 |
| **Single image** | 527 | 527 | 420 | 420 | 107 | 107 |  |
| **Multiple images** | 133 | 300 | 106 | 273 | 27 | 27 |  |
| **Benign** | 615 | 782 | 481 | 649 | 134 | 134 |  |
| **Malignant** | 45 | 45 | 45 | 45 | 0 | 0 |  |
| **Category IVc** | 481 | 619 | 380 | 518 | 101 | 101 | 0.566 |
| **Single image** | 414 | 414 | 327 | 327 | 87 | 87 |  |
| **Multiple images** | 67 | 205 | 53 | 191 | 14 | 14 |  |
| **Benign** | 413 | 551 | 312 | 450 | 101 | 101 |  |
| **Malignant** | 68 | 68 | 68 | 68 | 0 | 0 |  |
| **Category V** | 239 | 341 | 193 | 295 | 46 | 46 | 0.762 |
| **Single image** | 192 | 192 | 154 | 154 | 38 | 38 |  |
| **Multiple images** | 47 | 149 | 39 | 141 | 8 | 8 |  |
| **Benign** | 198 | 290 | 152 | 244 | 46 | 46 |  |
| **Malignant** | 41 | 51 | 41 | 51 | 0 | 0 |  |

## Supplementary Table 4. Comparison of Patient Demographics and Clinical Characteristics

| **Characteristic** | **Training Set** | **Validation Set** | **P value** |
| --- | --- | --- | --- |
| **Age, years** | 45.3 ± 12.7 | 45.8 ± 12.4 | 0.421§ |
| **Age groups** |  |  | 0.678 |
| **<40 years** | 612 (28.5%) | 147 (27.4%) |  |
| **40-49 years** | 758 (35.3%) | 195 (36.3%) |  |
| **50-59 years** | 536 (25.0%) | 132 (24.6%) |  |
| **≥60 years** | 242 (11.3%) | 63 (11.7%) |  |
| **Lesion size, mm** | 18.5 ± 11.2 | 18.9 ± 10.8 | 0.512§ |
| **Size categories** |  |  | 0.834 |
| **<10 mm** | 387 (18.0%) | 94 (17.5%) |  |
| **10-19 mm** | 1,032 (48.0%) | 261 (48.6%) |  |
| **20-29 mm** | 515 (24.0%) | 127 (23.7%) |  |
| **≥30 mm** | 214 (10.0%) | 55 (10.2%) |  |
| **Lesion location** |  |  | 0.912 |
| **Left breast** | 1,074 (50.0%) | 271 (50.5%) |  |
| **Right breast** | 1,074 (50.0%) | 266 (49.5%) |  |
| **Lesion depth** |  |  | 0.756 |
| **Superficial (<15 mm from skin)** | 645 (30.0%) | 158 (29.4%) |  |
| **Intermediate (15-30 mm)** | 1,074 (50.0%) | 274 (51.0%) |  |
| **Deep (>30 mm)** | 429 (20.0%) | 105 (19.6%) |  |

## Supplementary Table 5. Inter-Observer Segmentation Reliability Analysis

Inter-observer segmentation reliability was assessed on 50 randomly selected cases (1.9% of dataset). The two radiologists achieved excellent agreement, with an overall ICC of 0.87 (95% CI: 0.84-0.90) across all radiomics features. Shape features demonstrated the highest reliability (ICC = 0.93, 95% CI: 0.90-0.95), while texture features showed good to excellent agreement (ICC range: 0.82-0.86). The mean Dice similarity coefficient for spatial overlap was 0.88 (95% CI: 0.84-0.91), indicating high consistency in lesion boundary delineation.

(A) Intraclass Correlation Coefficients (ICC) for Radiomics Features

| **Feature Category** | **Number of** | **ICC (95% CI)** | | **Interpretation†** | **Features with** |
| --- | --- | --- | --- | --- | --- |
|  | **Features** | **Mean** | **Range** |  | **ICC ≥0.80** |
| **FIRST-ORDER STATISTICAL FEATURES** | | | | | |
| Overall first-order features | 18 | 0.89 (0.85-0.92) | 0.82-0.94 | Excellent | 18/18 (100%) |
| Energy | 1 | 0.91 (0.87-0.94) | - | Excellent | ✓ |
| Entropy | 1 | 0.88 (0.84-0.91) | - | Excellent | ✓ |
| Kurtosis | 1 | 0.85 (0.80-0.89) | - | Excellent | ✓ |
| Skewness | 1 | 0.87 (0.83-0.90) | - | Excellent | ✓ |
| Mean, Median, Variance | 3 | 0.92 (0.89-0.94) | 0.90-0.94 | Excellent | 46084 |
| Percentiles (10th, 90th) | 2 | 0.90 (0.86-0.93) | 0.88-0.92 | Excellent | 46055 |
| Range, Interquartile Range | 2 | 0.86 (0.82-0.90) | 0.84-0.88 | Excellent | 46055 |
| Others (MAD, RMS, etc.) | 7 | 0.89 (0.85-0.92) | 0.82-0.93 | Excellent | 46210 |
| **SHAPE FEATURES** | | | | | |
| Overall shape features | 14 | 0.93 (0.90-0.95) | 0.88-0.97 | Excellent | 14/14 (100%) |
| Volume (2D Area) | 1 | 0.95 (0.92-0.97) | - | Excellent | ✓ |
| Surface Area (Perimeter) | 1 | 0.94 (0.91-0.96) | - | Excellent | ✓ |
| Maximum Diameter | 1 | 0.96 (0.94-0.97) | - | Excellent | ✓ |
| Sphericity (Circularity) | 1 | 0.92 (0.89-0.94) | - | Excellent | ✓ |
| Compactness, Elongation | 2 | 0.91 (0.88-0.94) | 0.89-0.93 | Excellent | 46055 |
| Others (Flatness, etc.) | 8 | 0.93 (0.90-0.95) | 0.88-0.96 | Excellent | 46242 |
| **TEXTURE FEATURES** | | | | | |
| Gray-Level Co-occurrence Matrix (GLCM) | 24 | 0.86 (0.82-0.90) | 0.78-0.92 | Excellent | 22/24 (91.7%) |
| Contrast, Correlation | 2 | 0.88 (0.84-0.91) | 0.86-0.90 | Excellent | 46055 |
| Energy, Homogeneity | 2 | 0.89 (0.85-0.92) | 0.87-0.91 | Excellent | 46055 |
| Entropy, Dissimilarity | 2 | 0.85 (0.81-0.89) | 0.83-0.87 | Excellent | 46055 |
| Others (ASM, IDM, etc.) | 18 | 0.86 (0.81-0.90) | 0.78-0.92 | Excellent | 16/18 |
| Gray-Level Run Length Matrix (GLRLM) | 16 | 0.84 (0.79-0.88) | 0.76-0.90 | Excellent | 14/16 (87.5%) |
| Short/Long Run Emphasis | 2 | 0.87 (0.83-0.90) | 0.85-0.89 | Excellent | 46055 |
| Gray-Level Non-Uniformity | 2 | 0.85 (0.81-0.89) | 0.83-0.87 | Excellent | 46055 |
| Run Length Non-Uniformity | 2 | 0.83 (0.78-0.87) | 0.80-0.86 | Excellent | 46055 |
| Others (Run Percentage, etc.) | 10 | 0.83 (0.78-0.88) | 0.76-0.90 | Excellent | 46244 |
| Gray-Level Size Zone Matrix (GLSZM) | 16 | 0.82 (0.77-0.87) | 0.74-0.89 | Excellent | 13/16 (81.3%) |
| Small/Large Area Emphasis | 2 | 0.86 (0.82-0.89) | 0.84-0.88 | Excellent | 46055 |
| Gray-Level Variance | 2 | 0.84 (0.80-0.88) | 0.82-0.86 | Excellent | 46055 |
| Zone Percentage | 1 | 0.78 (0.72-0.83) | - | Good | - |
| Others | 11 | 0.81 (0.76-0.86) | 0.74-0.89 | Excellent | 46276 |
| Gray-Level Dependence Matrix (GLDM) | 14 | 0.85 (0.81-0.89) | 0.77-0.91 | Excellent | 12/14 (85.7%) |
| Small/Large Dependence Emphasis | 2 | 0.87 (0.83-0.90) | 0.85-0.89 | Excellent | 46055 |
| Gray-Level Non-Uniformity | 2 | 0.86 (0.82-0.89) | 0.84-0.88 | Excellent | 46055 |
| Dependence Variance | 1 | 0.83 (0.79-0.87) | - | Excellent | ✓ |
| Others | 9 | 0.84 (0.80-0.88) | 0.77-0.91 | Excellent | 46212 |
| **OVERALL SUMMARY** | | | | | |
| All Radiomics Features | 102 | 0.87 (0.84-0.90) | 0.74-0.97 | Excellent | 93/102 (91.2%) |

1. Spatial Overlap Metrics (Dice Similarity Coefficient)

| **Lesion Characteristic** | **Number of Cases** | **Dice Coefficient Mean (95% CI)** | **Range** | **Cases with Dice ≥0.80** |
| --- | --- | --- | --- | --- |
| Overall (All Cases) | 50 | 0.88 (0.84-0.91) | 0.72-0.96 | 45/50 (90.0%) |
| **By Malignancy Status** | | | | |
| Benign lesions | 25 | 0.90 (0.86-0.93) | 0.78-0.96 | 24/25 (96.0%) |
| Malignant lesions | 25 | 0.86 (0.81-0.90) | 0.72-0.94 | 21/25 (84.0%) |
| **By BI-RADS Category** | | | | |
| BI-RADS 2 | 5 | 0.92 (0.88-0.95) | 0.87-0.96 | 5/5 (100%) |
| BI-RADS 3 | 10 | 0.91 (0.87-0.94) | 0.82-0.95 | 10/10 (100%) |
| BI-RADS 4a | 10 | 0.89 (0.85-0.92) | 0.78-0.94 | 9/10 (90.0%) |
| BI-RADS 4b | 10 | 0.86 (0.81-0.90) | 0.75-0.92 | 8/10 (80.0%) |
| BI-RADS 4c | 8 | 0.84 (0.78-0.89) | 0.72-0.91 | 6/8 (75.0%) |
| BI-RADS 5 | 7 | 0.85 (0.79-0.90) | 0.73-0.93 | 6/7 (85.7%) |
| **By Lesion Size** | | | | |
| Small (<10 mm) | 8 | 0.82 (0.76-0.87) | 0.72-0.89 | 6/8 (75.0%) |
| Medium (10-20 mm) | 24 | 0.89 (0.85-0.92) | 0.78-0.96 | 22/24 (91.7%) |
| Large (>20 mm) | 18 | 0.90 (0.86-0.93) | 0.81-0.95 | 17/18 (94.4%) |
| **By Margin Characteristics** | | | | |
| Circumscribed margins | 20 | 0.92 (0.89-0.94) | 0.84-0.96 | 20/20 (100%) |
| Non-circumscribed margins | 30 | 0.85 (0.81-0.89) | 0.72-0.94 | 25/30 (83.3%) |

(c) Cases Requiring Consensus Review

| **Discrepancy Reason** | **Number of Cases** | **Percentage** | **Initial Dice Coefficient** | **Final Dice After Consensus** |
| --- | --- | --- | --- | --- |
| Irregular/spiculated margins | 3 | 6.0% | 0.74 (0.72-0.76) | 0.89 (0.86-0.91) |
| Posterior acoustic shadowing | 1 | 2.0% | 0.75 | 0.87 |
| Ill-defined boundaries | 1 | 2.0% | 0.73 | 0.85 |
| Total cases requiring consensus | 5 | 10.0% | 0.74 (0.72-0.76) | 0.87 (0.85-0.91) |
| Cases with acceptable agreement (Dice ≥0.80) | 45 | 90.0% | 0.90 (0.87-0.93) | / |

**Abbreviations:** ICC, intraclass correlation coefficient; CI, confidence interval; GLCM, gray-level co-occurrence matrix; GLRLM, gray-level run length matrix; GLSZM, gray-level size zone matrix; GLDM, gray-level dependence matrix; BI-RADS, Breast Imaging Reporting and Data System; ROI, region of interest; MAD, mean absolute deviation; RMS, root mean square; ASM, angular second moment; IDM, inverse difference moment.
